# Integrated Transcriptomic Analysis Reveals Distinct Molecular and Immune Microenvironment Features of Peutz–Jeghers Syndrome-Associated Lobular Endocervical Glandular Hyperplasia and Gastric-Type Endocervical Adenocarcinoma

**DOI:** 10.3390/biomedicines14102197

**Published:** 2026-09-28

**Authors:** Xiaoqin Gong, Yiqing Chen, Anqi Jiang, Yingfei Ye, Hui Wang, Ranran Chai, Jing Xu, Guanqin Jin, Hongwei Zhang, Jinyi Tong, Yu Kang

**Affiliations:** 1 Department of Obstetrics and Gynecology, Shanghai Key Laboratory of Reproduction and Development, Shanghai Key Laboratory of Female Reproductive Endocrine-Related Diseases, Obstetrics and Gynecology Hospital of Fudan University, Shanghai 200090, China; 13554157866@163.com (X.G.); 18189361906@163.com (Y.C.); jaq0413@163.com (A.J.); yeyingfei2024@126.com (Y.Y.); chairanran7664@fckyy.org.cn (R.C.); doctor_xj@163.com (J.X.); zhanghongwei0824@aliyun.com (H.Z.); 2 Phase One Clinical Trials Unit, Obstetrics and Gynecology Hospital of Fudan University, Shanghai 200090, China; wanghui7611@fckyy.org.cn (H.W.); jinguanqin7889@fckyy.org.cn (G.J.); 3 Department of Gynecology, Affiliated Hangzhou First People’s Hospital, School of Medicine, Westlake University, Hangzhou 310006, China

**Keywords:** Peutz–Jeghers syndrome, gastric-type endocervical adenocarcinoma, lobular endocervical glandular hyperplasia, transcriptomic analysis, weighted gene co-expression network analysis, immune microenvironment

## Abstract

**Background:** Gastric-type endocervical adenocarcinoma (GAS) is an aggressive, human papillomavirus-independent cervical adenocarcinoma, whereas lobular endocervical glandular hyperplasia (LEGH) is considered a potential precursor. Molecular and immune microenvironment differences between Peutz–Jeghers syndrome-associated LEGH (PJS-LEGH) and GAS (PJS-GAS) remain poorly characterized. This study aimed to characterize these differences through integrated transcriptomic analysis. **Methods:** Bulk RNA sequencing was performed on tissues from 7 PJS-LEGH and 8 PJS-GAS cases. Differential expression and functional enrichment analyses were integrated with weighted gene co-expression network analysis (WGCNA), molecular interaction network analysis, and machine learning. Immune-related transcriptional patterns were assessed using single-sample gene set enrichment analysis (ssGSEA) and xCell, with CD45/CD4 multiplex immunofluorescence for preliminary tissue-level assessment. **Results:** We identified 1794 differentially expressed genes, including 976 upregulated and 818 downregulated genes in PJS-GAS. Functional and network analyses highlighted differences involving ciliary biology, epithelial differentiation, chemokine signaling, and immune regulation. ssGSEA revealed higher scores for selected T-cell, dendritic-cell, monocyte, and myeloid-derived suppressor cell signatures in PJS-GAS. xCell provided complementary evidence of higher scores for selected immune-cell populations, together with activation- and inhibition-associated transcriptional signatures. Integrated analyses converged on *PTPRC*, encoding the pan-leukocyte marker CD45, as a prominent leukocyte-associated feature of PJS-GAS. Multiplex immunofluorescence showed higher CD45^+^ and CD4^+^ cell densities in the sampled PJS-GAS tissues. **Conclusions:** This study provides the first transcriptomic comparison of PJS-LEGH and PJS-GAS and identifies distinct molecular features and increased immune-cell-associated transcriptional signatures with coexisting activation- and inhibition-associated expression patterns in PJS-GAS.

## 1. Introduction

Cervical cancer is one of the most common malignancies among women. Its major histologic types include cervical squamous cell carcinoma and cervical adenocarcinoma, and the relative incidence of cervical adenocarcinoma has increased in recent years [1,2]. Gastric-type endocervical adenocarcinoma (GAS) is the most common subtype of HPV-independent cervical adenocarcinoma, accounting for approximately 10% of all cervical adenocarcinomas [3,4]. GAS is often diagnosed at an advanced stage and is characterized by aggressive biological behavior, including deep stromal invasion, lymphovascular invasion, and distant metastasis. It also shows limited response to conventional chemoradiotherapy and is associated with poor prognosis [5,6,7,8]. Notably, approximately 10% of GAS cases are associated with Peutz–Jeghers syndrome (PJS) [9]. Patients with PJS-associated GAS (PJS-GAS) may present at a young age and have poor clinical outcomes [10], suggesting that PJS-GAS may have distinct biological features. However, because of its rarity and unique genetic background, the mechanisms underlying the development of PJS-GAS remain poorly understood.

Lobular endocervical glandular hyperplasia (LEGH) is an important lesion within the gastric-type differentiation spectrum of the cervix and has been proposed as a potential precursor lesion of GAS in some cases. Previous studies have supported a possible relationship between LEGH and GAS at the morphologic, immunophenotypic, and genetic levels. For example, both lesions may express gastric-type markers, such as MUC6 and HIK1083 [11]. Molecular studies have reported clonal relationships between LEGH and associated carcinomas, with *STK11* alterations implicated in malignant progression [12] and a shared *GNAS* variant identified in adjacent LEGH and GAS [13]. However, current studies have mainly focused on histopathologic morphology and genomic alterations, whereas transcriptomic and immune microenvironmental differences between PJS-LEGH and PJS-GAS have not been investigated to date.

Transcriptomic analyses have provided important insights into the biological features of GAS. Vasseur et al. reported that GAS exhibits a gene expression profile distinct from that of usual-type endocervical adenocarcinoma and suggested that tight junction disruption may be associated with the metastatic potential of mucinous endocervical adenocarcinomas, including GAS [14]. Another study integrating whole-transcriptome sequencing and metabolomic profiling showed sustained transcriptional suppression of *TP53*-related pathways in GAS [15]. Regarding the immune microenvironment, a recent study using bulk RNA sequencing and multiplex immunofluorescence demonstrated that GAS may exhibit reduced T-cell infiltration, macrophage enrichment, and tertiary lymphoid structure formation [16]. These findings suggest that GAS development involves not only tumor-intrinsic molecular abnormalities but also alterations in the immune microenvironment. However, existing studies have primarily characterized GAS in comparison with usual-type endocervical adenocarcinoma or other cervical cancer subtypes, limiting the extent to which these findings can inform the biology of PJS-associated gastric-type glandular lesions.

Given the limited molecular characterization of PJS-associated gastric-type cervical glandular lesions, this study aimed to characterize the molecular and immune microenvironmental differences between PJS-LEGH and PJS-GAS through integrated transcriptomic analysis. This is the first comparative transcriptomic study of PJS-LEGH and PJS-GAS, providing molecular insights into these rare lesions and a basis for further investigation of the molecular characteristics and immune microenvironment associated with PJS-GAS.

## 2. Materials and Methods

### 2.1. Clinical Samples

Patients were diagnosed with Peutz–Jeghers syndrome (PJS) according to established clinical criteria and/or germline *STK11* testing [17]. A clinical diagnosis of PJS was made when any of the following criteria was met: (1) two or more histologically confirmed Peutz–Jeghers-type hamartomatous polyps; (2) any number of Peutz–Jeghers-type polyps in an individual with a family history of PJS, defined as at least one first-degree relative with PJS; (3) characteristic mucocutaneous pigmentation in an individual with a family history of PJS; or (4) characteristic mucocutaneous pigmentation together with any number of Peutz–Jeghers-type polyps. Germline *STK11* testing was used to support or confirm the diagnosis when clinical criteria were not fully met.

This study included fresh-frozen tissue samples from 7 patients with pathologically confirmed PJS-LEGH and 8 patients with pathologically confirmed PJS-GAS. Samples were included based on definitive histopathologic diagnosis, sufficient tissue availability, adequate RNA quality, and complete clinicopathologic information; samples failing expression-profiling quality control were excluded. HPV status was obtained from routine clinical screening records at the Obstetrics and Gynecology Hospital of Fudan University. Testing was performed using the Roche cobas 4800 HPV system (Roche Molecular Systems, Pleasanton, CA, USA). The study was approved by the Ethics Committee of the Obstetrics and Gynecology Hospital of Fudan University (approval No. 2022-133; 31 August 2022).

### 2.2. RNA Sequencing and Data Preprocessing

Total RNA was extracted from tissue samples using TRIzol™ Reagent (Invitrogen, Carlsbad, CA, USA; Cat. No. 15596026). RNA purity was evaluated based on OD260/280 and OD260/230 ratios, and RNA integrity was assessed using an Agilent 2100 Bioanalyzer (Agilent Technologies, Santa Clara, CA, USA). Qualified RNA samples were subjected to poly(A) mRNA enrichment, fragmentation, cDNA synthesis, adaptor ligation, and PCR amplification for library construction, followed by high-throughput sequencing. After filtering out lowly expressed genes, raw gene count matrices were used for differential expression analysis, whereas normalized expression values were used for visualization and downstream analyses [18].

### 2.3. Principal Component Analysis

Principal component analysis (PCA) was performed to evaluate overall transcriptomic differences between PJS-associated LEGH and GAS samples. Normalized expression values were used for PCA. Genes were ranked according to their expression variance across all samples, and the top 1000 highly variable genes were selected for PCA visualization.

### 2.4. Identification of Differentially Expressed Genes (DEGs) and Functional Enrichment Analysis

Differential expression analysis was performed using DESeq2 (version 1.50.2) based on the raw gene count matrix. Genes with an absolute log_2_ fold change > 1 and a nominal *p*-value < 0.05 were retained for exploratory downstream enrichment and network analyses. This permissive threshold was used to retain potentially relevant expression changes for hypothesis generation.

Gene Ontology (GO) enrichment and Kyoto Encyclopedia of Genes and Genomes (KEGG) pathway analyses were performed using the clusterProfiler package (version 4.18.4) in R to characterize the biological functions and pathways associated with DEGs. GO terms were classified into three categories: biological process (BP), cellular component (CC), and molecular function (MF). GO terms and KEGG pathways with an adjusted *p*-value < 0.05 were considered significantly enriched.

### 2.5. Weighted Gene Co-Expression Network Analysis

Weighted gene co-expression network analysis (WGCNA) was performed using the WGCNA package in R. After preprocessing, 18,342 genes were retained for network construction. The soft-thresholding power was selected using the pickSoftThreshold function, and a power of 5 was chosen because the scale-free topology fitting index exceeded 0.85. Module eigengenes were calculated, and Pearson correlation analysis was used to evaluate associations between modules and phenotypes. Modules showing significant positive correlations with GAS (*p* < 0.05) were considered GAS-associated modules. Six such modules, comprising 2009 genes in total, were selected for subsequent analyses. Given the limited sample size (*n* = 15), which reflects the rarity of this disease spectrum, WGCNA was used as an exploratory analysis, and module–trait associations were interpreted as hypothesis-generating rather than definitive network inferences.

### 2.6. Construction of the PJS-Associated Gene Set

PJS-associated genes were retrieved from the GeneCards database (https://www.genecards.org, accessed on 16 August 2025) using the keyword “Peutz–Jeghers syndrome”. GeneCards integrates information from multiple biological databases and provides an evidence-weighted view of gene–disease and gene–function relationships [19]. The relevance score was used to define a literature-informed candidate set. In this study, protein-coding genes with a relevance score > 5 were retained based on previously published GeneCards-based studies [20,21]. A total of 942 PJS-associated genes were included in the final gene set and used for subsequent intersection analysis with DEGs and GAS-associated WGCNA module genes.

### 2.7. Protein–Protein Interaction (PPI) Network Analysis and Exploratory Machine-Learning-Based Candidate Gene Prioritization

The overlapping candidate genes were imported into STRING to construct a protein–protein interaction (PPI) network using a minimum combined score of 0.4, without adding interactors. The resulting network was then exported and visualized using Cytoscape (version 3.10.4). The top 10 genes ranked by node degree were retained for exploratory candidate prioritization, with ties resolved alphabetically by gene symbol.

To further prioritize candidate genes, least absolute shrinkage and selection operator (LASSO) regression, random forest analysis, and PPI network topology were integrated. LASSO regression was performed using the glmnet package (version 4.1-10) in R, with GAS/LEGH status as the dependent variable and candidate-gene expression levels as the independent variables. Five-fold cross-validation was used to select the penalty parameter, and genes with nonzero coefficients at lambda.1se were retained. Random forest analysis was performed using the randomForest package (version 4.7-1.2) in R with 1000 trees. The out-of-bag error curve was examined as an internal diagnostic of the random forest analysis. Genes were ranked according to MeanDecreaseGini, and those with MeanDecreaseGini > 0.2 were retained as important features. Candidate genes jointly identified by PPI network topology, LASSO regression, and random forest analysis were retained for further exploratory analysis. To assess the sensitivity of candidate prioritization to the GeneCards relevance-score cutoff, we repeated the intersection and downstream LASSO, random forest, and PPI analyses at cutoffs from >5 to >10 in increments of 1, while keeping all other filtering criteria and model settings unchanged.

### 2.8. Immune Infiltration Analysis

The single-sample gene set enrichment analysis (ssGSEA) algorithm implemented in the GSVA R package (version 2.4.7) was used to calculate enrichment scores for immune-cell-associated and immune-function-related gene signatures in each sample. Scores for 28 immune cell types were calculated using previously reported immune cell signature gene sets [22]. In addition, 13 immune-related functions and 16 immune cell features were evaluated based on predefined immune gene sets [23]. The xCell algorithm (version 1.1.0) was additionally applied as a complementary computational approach to assess the consistency of immune infiltration patterns [24]. Antitumor and protumor scores were calculated by summing the across-sample z-standardized ssGSEA scores of the selected signatures.

To explore potential stage-related variation in immune signatures, associations between International Federation of Gynecology and Obstetrics (FIGO) stage and ssGSEA/xCell enrichment scores were examined within the eight PJS-GAS cases. FIGO stage was treated as an ordered categorical variable (IA1 < IB2 < IB3 < IIB < IIIC1 < IVB). A supplementary exploratory comparison was performed between FIGO stages I–IIA and IIB–IVB (*n* = 4 each), using the IIA/IIB boundary adopted by Chen et al. [25].

### 2.9. Multiplex Immunofluorescence Staining and Image Analysis

For tissue-level assessment, multiplex immunofluorescence was performed on available formalin-fixed, paraffin-embedded (FFPE) sections from 3 PJS-LEGH and 3 PJS-GAS cases. A subset of cases with adequate staining quality and morphologically representative lesion tissue was selected, independently of *PTPRC* expression or immune-infiltration status. Two cases per group were also included in the RNA-sequencing cohort, whereas one additional case per group was assessed only by multiplex immunofluorescence. The included samples and their overlap with the RNA-sequencing cohort are listed in Table S1. Multiplex immunofluorescence was used as a preliminary tissue-level assessment of CD45^+^ and CD4^+^ immune-cell infiltration patterns.

Multiplex immunofluorescence staining was performed using a tyramide signal amplification (TSA)-based three-marker, four-color protocol targeting pan-cytokeratin (PanCK), CD45/PTPRC, and CD4, with DAPI nuclear counterstaining. FFPE tissue sections were deparaffinized, rehydrated, and subjected to heat-induced antigen retrieval for 15 min. After blocking endogenous peroxidase activity and nonspecific binding, sections were sequentially incubated overnight at 4 °C with primary antibodies against PanCK (Servicebio, Wuhan, China; GB122053; 1:5000), CD45/PTPRC (Servicebio, Wuhan, China; GB115428; 1:3000), and CD4 (Servicebio, Wuhan, China; GB13588; 1:2000). Corresponding horseradish peroxidase (HRP)-conjugated polymer secondary antibodies (Servicebio, Wuhan, China) were applied for 50 min at room temperature, followed by TSA fluorophores (Servicebio, Wuhan, China) for 10 min. PanCK, CD45/PTPRC, and CD4 were visualized using TSA 488, TSA 555, and TSA 647, respectively. Antibody elution was performed between staining cycles. Nuclei were counterstained with DAPI, tissue autofluorescence was quenched, and slides were mounted with antifade mounting medium. Images were acquired using a confocal microscope (Leica DMi8, Leica Microsystems, Wetzlar, Germany).

Quantitative image analysis was performed using QuPath v0.7.0. Original multichannel LIF files were imported through Bio-Formats (version 8.4.0), and channels were assigned as DAPI, PanCK, CD45/PTPRC, and CD4. Cells were detected based on DAPI-stained nuclei, and marker positivity was determined using fixed fluorescence intensity thresholds. CD45^+^ cells were defined as cells positive for CD45/PTPRC staining, whereas CD4^+^ cells were defined as cells positive for both CD45/PTPRC and CD4 staining. PanCK staining was used to delineate PanCK^+^ epithelial/tumor regions and PanCK^−^ stromal regions, and cells were assigned to these compartments according to the locations of their cell centroids. Cell densities were calculated as cells/mm^2^ for the whole field and separately within PanCK-defined tissue compartments. The percentage of CD45^+^CD4^+^ cells among total CD45^+^ cells was also calculated. For each case, measurements from three fields of view were averaged to obtain a single patient-level value for subsequent between-group statistical comparison.

### 2.10. Statistical Analysis

Statistical analyses were performed using R (version 4.5.2) and Python (version 3.14.5) with SciPy (version 1.18.0). Continuous variables between two groups were compared using Student’s t-test or the Wilcoxon rank-sum test, as appropriate. For immunofluorescence measurements, normality was assessed using Shapiro–Wilk tests within each group. Exploratory Student’s *t*-tests were supplemented with exact permutation tests based on the absolute between-group difference in patient-level means across all 20 group assignments; *p*-values were unadjusted for multiple comparisons. Given the modest group sizes (PJS-GAS, *n* = 8; PJS-LEGH, *n* = 7), effect sizes were additionally reported for ssGSEA-derived immune-score comparisons, including Cliff’s delta with bootstrap-derived 95% confidence intervals and Hedges’ g as a bias-corrected standardized mean difference. Spearman correlation was used for *PTPRC*–immune-score and FIGO stage–immune-score associations; Pearson correlation was used between antitumor and protumor immune scores. *PTPRC* correlation annotations used unadjusted two-sided *p*-values. Stage-group comparisons used Mann–Whitney tests, with Cliff’s delta as the effect size. Both stage-related analyses used two-sided exact permutation *p*-values and Benjamini–Hochberg correction separately within the ssGSEA and xCell score panels. All tests were two-sided, and *p* < 0.05 was considered statistically significant unless otherwise specified. For functional enrichment analyses, *p*-values were adjusted using the Benjamini–Hochberg method where applicable. Figures were generated using R packages, including ggplot2 (version 4.0.2), pheatmap (version 1.0.13), and ComplexHeatmap (version 2.26.1), Python with Matplotlib (version 3.11.0), and GraphPad Prism (version 10.1.2; GraphPad Software, Boston, MA, USA). Network visualization was performed using Cytoscape.

## 3. Results

### 3.1. Transcriptomic Profiling Reveals Distinct Gene-Expression Patterns Between PJS-LEGH and PJS-GAS

The clinicopathologic characteristics of the included patients are summarized in Table 1. This study included 7 patients with PJS-LEGH and 8 patients with PJS-GAS. The age at diagnosis ranged from 18 to 38 years in the PJS-LEGH group and from 26 to 44 years in the PJS-GAS group. All patients were HPV-negative. Watery vaginal discharge was present in all 8 PJS-GAS cases (100%) and in 5 of 7 PJS-LEGH cases (71.4%), whereas 2 of 7 patients with PJS-LEGH (28.6%) were asymptomatic. Germline *STK11* mutations were detected in 8/8 PJS-GAS cases (100%) and 6/7 PJS-LEGH cases (85.7%). The PJS-GAS cases encompassed FIGO stages IA1 to IVB.

**Figure 1:**
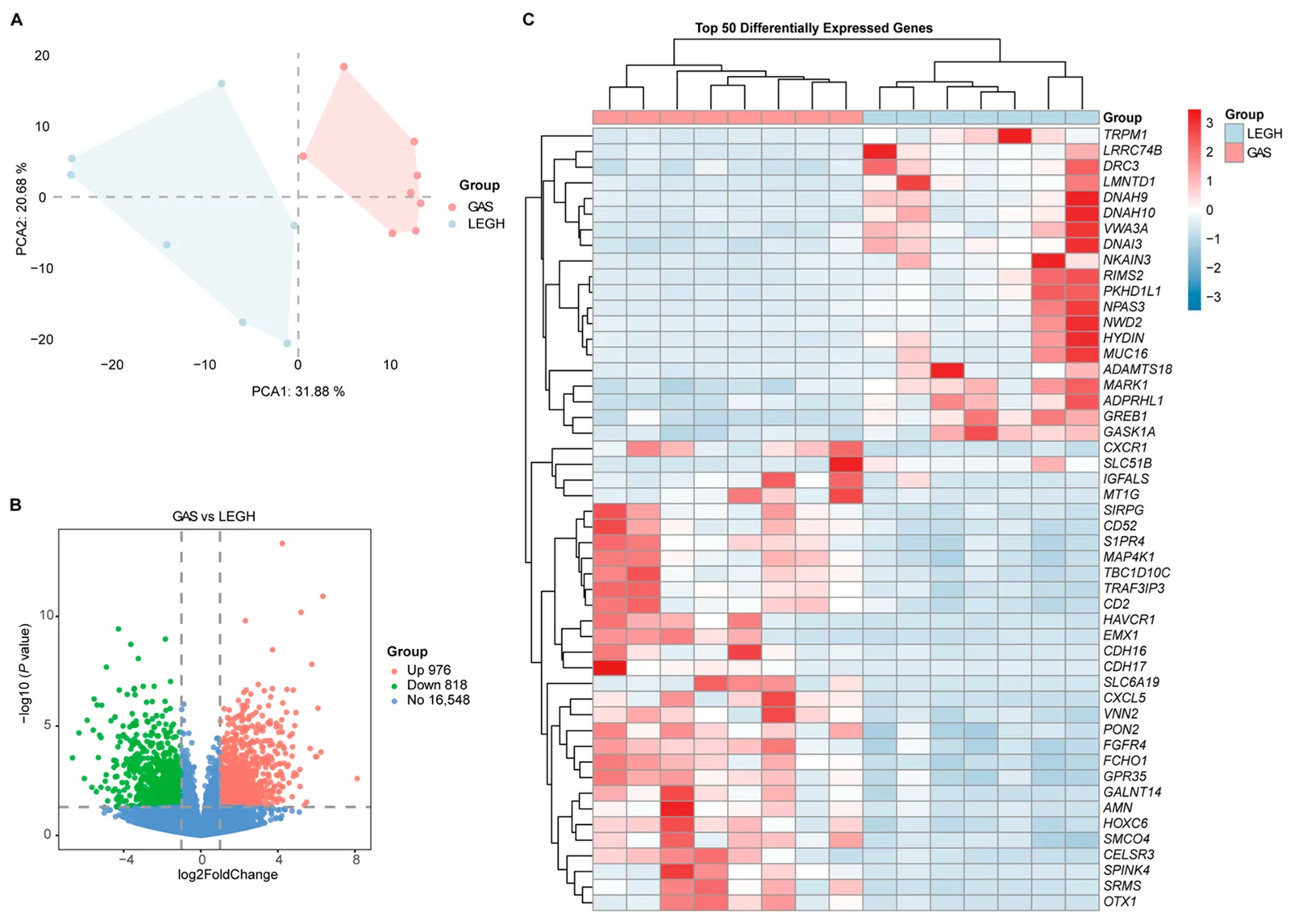
Identification of differentially expressed genes between PJS-LEGH and PJS-GAS. (**A**) Principal component analysis (PCA) plot based on the top 1000 most variable genes. (**B**) Volcano plot of differentially expressed genes in PJS-GAS versus PJS-LEGH. Red dots indicate upregulated genes, green dots indicate downregulated genes, and blue dots indicate non-significant genes. (**C**) Heatmap showing the expression profiles of the top 50 differentially expressed genes between PJS-LEGH and PJS-GAS.

PCA based on the top 1000 most variable genes showed clear separation between the two groups (Figure 1A). Differential expression analysis identified 1794 DEGs in PJS-GAS versus PJS-LEGH using |log_2_ fold change| > 1 and nominal *p* < 0.05, including 976 upregulated and 818 downregulated genes (Figure 1B). Among these DEGs, *SPINK4*, *SLC6A19*, *OTX1*, and the chemokine-related gene *CXCL5* were significantly upregulated in PJS-GAS, whereas *TRPM1*, *NPAS3*, *LMNTD1*, and *NWD2* were significantly downregulated. The heatmap of the top 50 DEGs further illustrated the expression differences between PJS-LEGH and PJS-GAS (Figure 1C), suggesting marked transcriptomic differences between the two groups.

### 3.2. Functonal Enrichment Highlights Cilium Movement, Epithelial Differentiation, and Immune-Inflammatory Pathways

**Figure 2:**
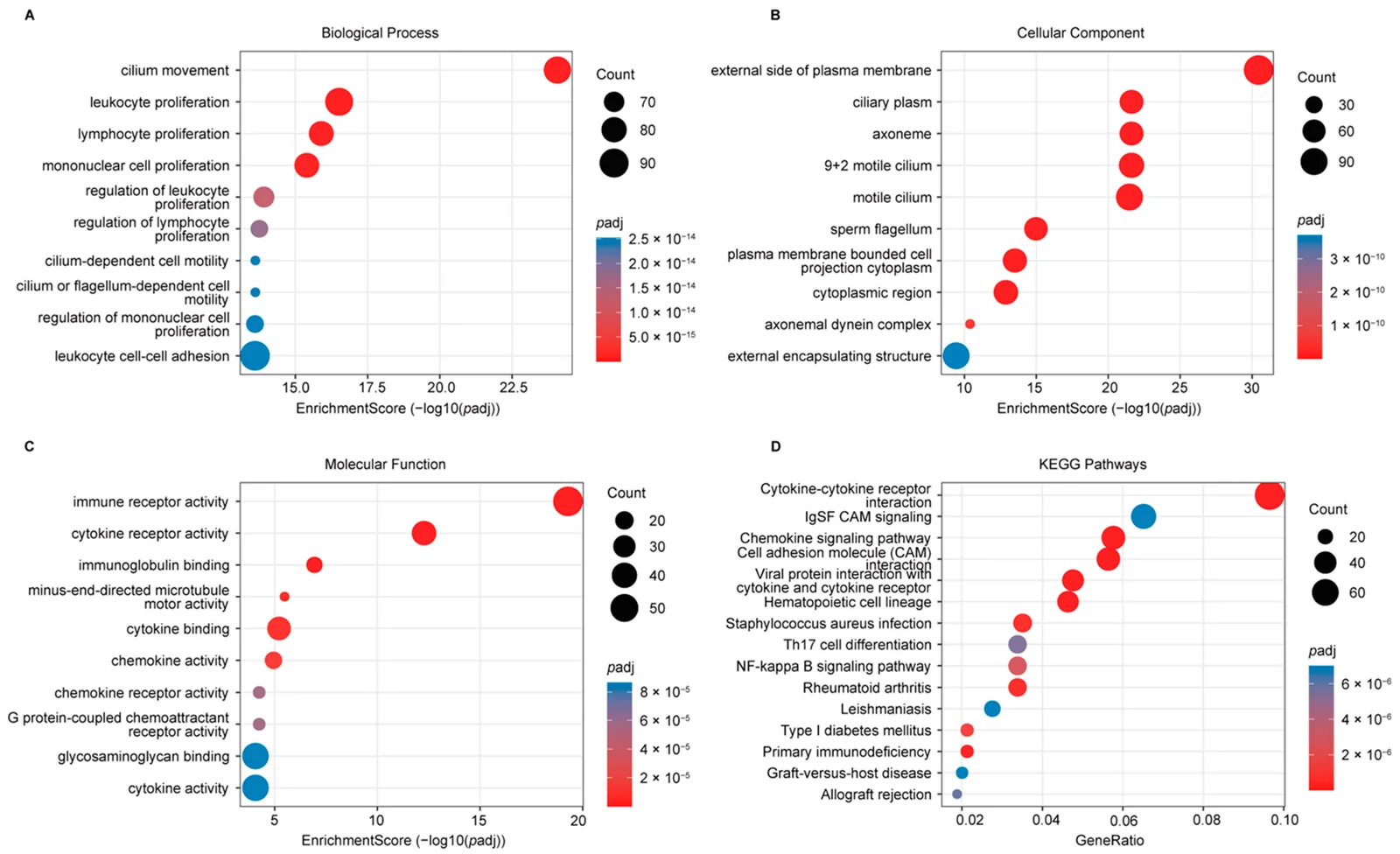
Functional enrichment analysis of differentially expressed genes between PJS-LEGH and PJS-GAS. (**A**) Bubble plot of Gene Ontology (GO) biological process enrichment analysis. (**B**) Bubble plot of GO cellular component enrichment analysis. (**C**) Bubble plot of GO molecular function enrichment analysis. (**D**) Bubble plot of Kyoto Encyclopedia of Genes and Genomes (KEGG) pathway enrichment analysis. In all panels, bubble size represents gene count, and color represents the Benjamini–Hochberg-adjusted *p*-value.

To explore the biological functions associated with the DEGs between PJS-LEGH and PJS-GAS, GO and KEGG enrichment analyses were performed. GO biological process analysis showed that these DEGs were mainly enriched in cilium movement, leukocyte proliferation, lymphocyte proliferation, mononuclear cell proliferation, and leukocyte cell–cell adhesion (Figure 2A). GO cellular component analysis revealed enrichment in ciliary structures, including ciliary plasm, axoneme, 9 + 2 motile cilium, and motile cilium (Figure 2B). GO molecular function analysis showed enrichment in immune receptor activity, cytokine receptor activity, cytokine activity, chemokine activity, and glycosaminoglycan binding (Figure 2C). KEGG pathway analysis further indicated that the DEGs were enriched in cytokine-cytokine receptor interaction, chemokine signaling pathway, cell adhesion molecules, Th17 cell differentiation, and NF-κB signaling pathway (Figure 2D). In addition, cilia-related DEGs showed distinct enrichment and expression patterns between PJS-LEGH and PJS-GAS samples (Figure S1A,B), and epithelial differentiation-related DEGs also exhibited dysregulated expression patterns between the two groups (Figure S2A,B). These results highlight transcriptional differences between PJS-GAS and PJS-LEGH involving genes associated with ciliary processes, epithelial differentiation, and immune-inflammatory pathways, including chemokine signaling.

### 3.3. WGCNA Identifies Phenotype-Associated Co-Expression Modules in LEGH and GAS

**Figure 3:**
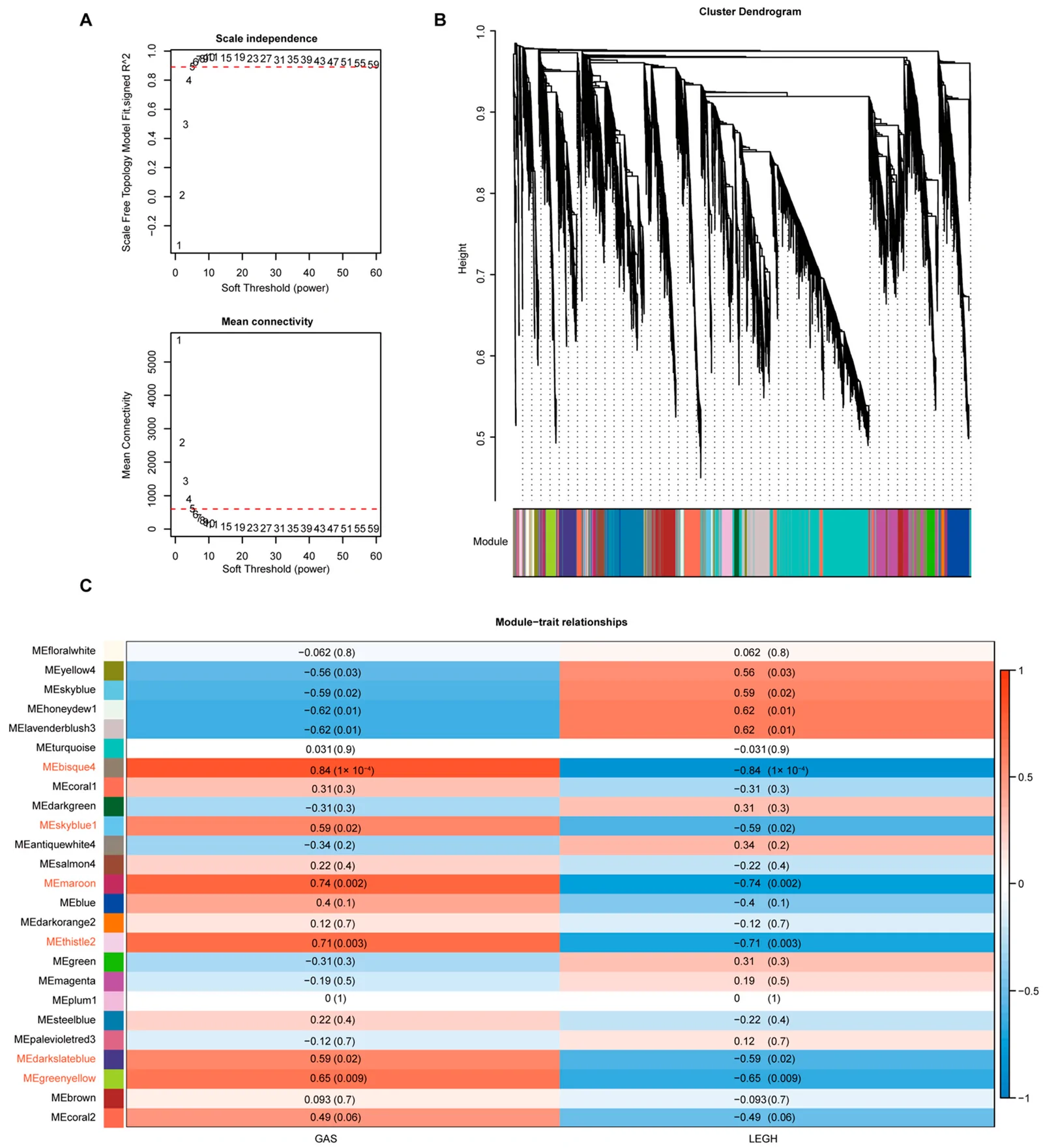
Identification of co-expression modules associated with PJS-LEGH and PJS-GAS phenotypes by weighted gene co-expression network analysis (WGCNA). (**A**) Determination of the soft-thresholding power. (**B**) Gene dendrogram and module assignment, with different colors representing distinct co-expression modules. (**C**) Module–trait relationship heatmap showing correlations between module eigengenes and PJS-LEGH or PJS-GAS phenotypes. Numbers in each cell indicate the correlation coefficient, with the corresponding *p*-value shown in parentheses; red and blue indicate positive and negative correlations, respectively.

Exploratory WGCNA was performed to identify co-expression modules associated with PJS-GAS and PJS-LEGH phenotypes. A soft-thresholding power of 5 was selected to construct a scale-free network (Figure 3A). Hierarchical clustering classified genes into multiple co-expression modules (Figure 3B). Module-trait correlation analysis showed that several modules were significantly associated with PJS-GAS or PJS-LEGH phenotypes, indicating distinct co-expression patterns between the two groups (Figure 3C). Among them, six modules showing significant positive correlations with PJS-GAS (*p* < 0.05), comprising 2009 genes in total, were selected for further analysis.

### 3.4. Integrated Candidate Genes Are Enriched in Immune-Related Biological Processes

**Figure 4:**
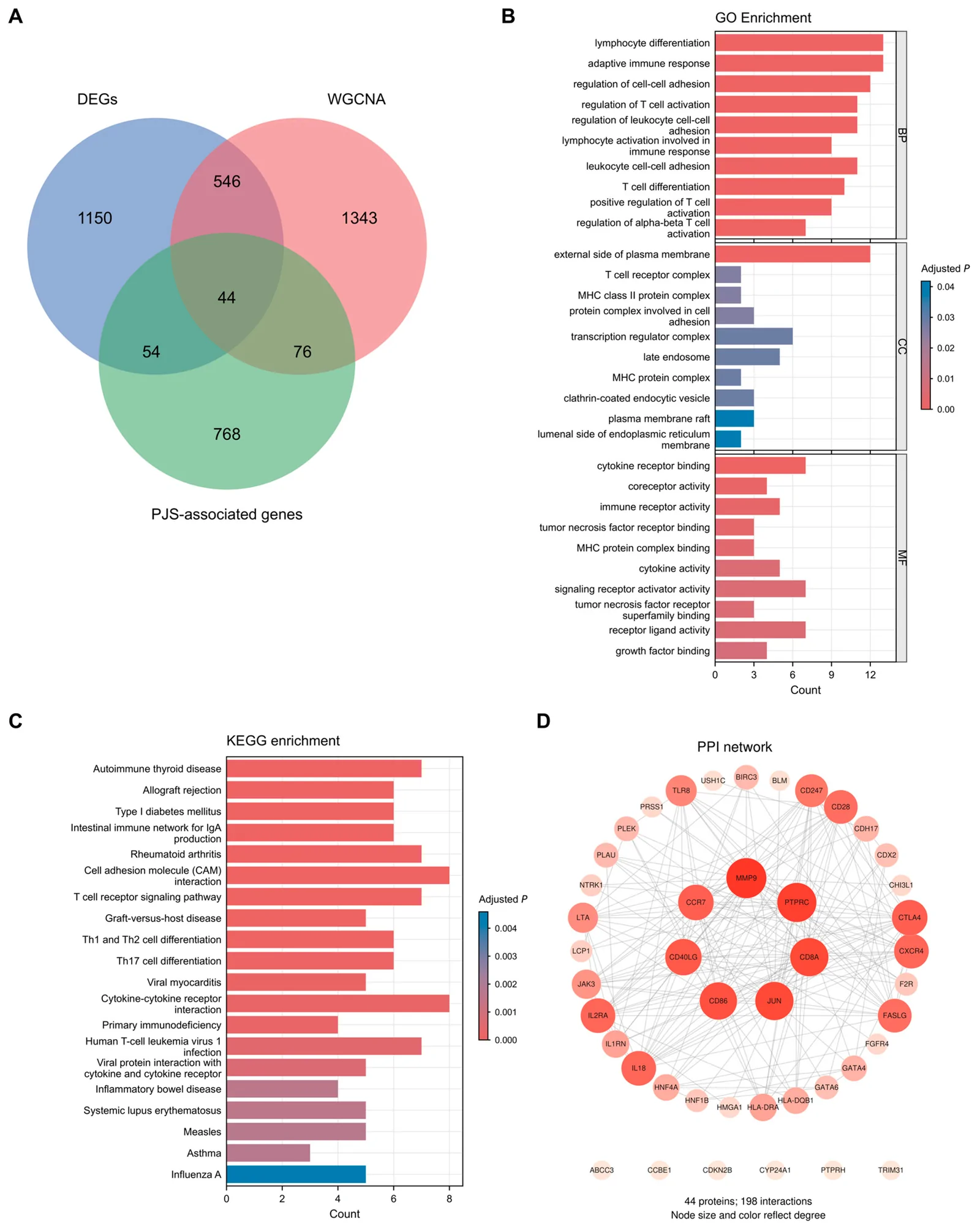
Intersecting candidate genes are mainly enriched in immune-related processes. (**A**) Venn diagram showing the overlap among differentially expressed genes (DEGs), genes from GAS-associated WGCNA modules, and PJS-associated genes. (**B**) Gene Ontology (GO) enrichment analysis of intersecting candidate genes. (**C**) Kyoto Encyclopedia of Genes and Genomes (KEGG) pathway enrichment analysis of intersecting candidate genes. In (**B**,**C**), bar length indicates gene count and color indicates Benjamini–Hochberg-adjusted *p*-values. (**D**) Protein–protein interaction (PPI) network of intersecting candidate genes; node size and color reflect degree.

To further identify genes potentially involved in the molecular differences between PJS-GAS and PJS-LEGH, DEGs, GAS-associated WGCNA module genes, and PJS-associated genes were intersected. A total of 44 candidate genes were identified (Figure 4A). GO enrichment analysis highlighted lymphocyte differentiation, adaptive immune response, regulation of T-cell activation, cytokine receptor binding, and immune receptor activity (Figure 4B). KEGG enrichment analysis identified pathways including cytokine–cytokine receptor interaction, T-cell receptor signaling, and Th17-cell differentiation (Figure 4C). The STRING-derived network comprised 44 proteins and 198 interactions, with six proteins having no qualifying interactions. Highly connected proteins included MMP9, PTPRC, CD8A, JUN, and CD86 (Figure 4D). These results highlight immune-related functional annotations within the integrated candidate set.

### 3.5. Integrated Machine Learning and Network Analyses Converge on PTPRC as a Shared Immune-Related Feature

**Figure 5:**
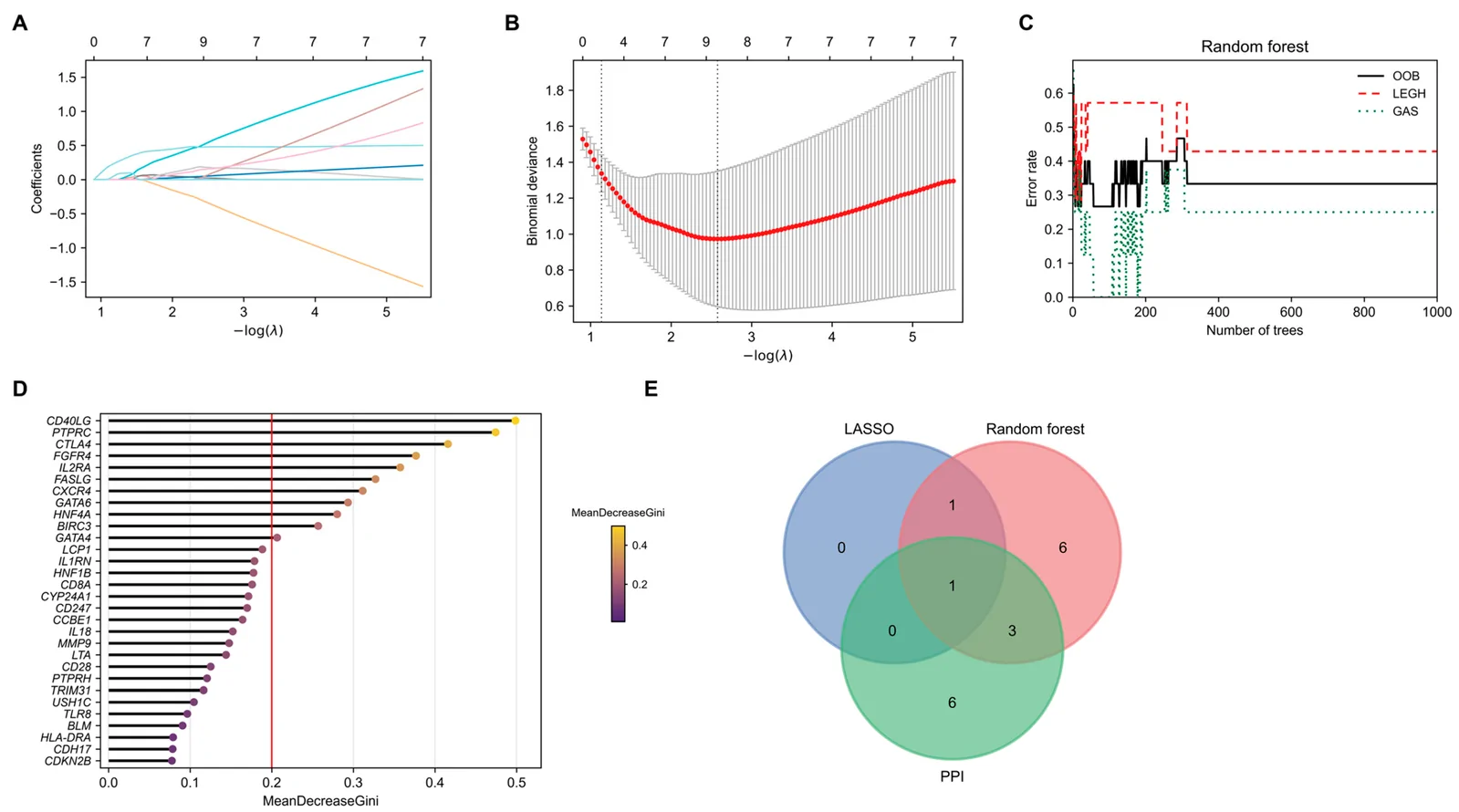
Exploratory prioritization of candidate features using least absolute shrinkage and selection operator (LASSO), random forest, and protein–protein interaction (PPI) network analysis. (**A**) LASSO coefficient paths; different colors distinguish individual candidate genes. (**B**) Cross-validation curve used to select the penalty parameter λ. Red points indicate the mean cross-validated binomial deviance, and gray error bars represent ±1 standard error. The left and right vertical dotted lines indicate λ_1_se and λmin, respectively; λ_1_se was used for feature selection. Numbers along the upper axes in panels A and B indicate the number of nonzero coefficients at the corresponding λ values. (**C**) Out-of-bag (OOB) and class-specific error curves of the random forest model. The black solid line indicates the overall OOB error, the red dashed line the LEGH-specific error, and the green dotted line the GAS-specific error. (**D**) Top 30 random forest features ranked by MeanDecreaseGini. Point position and color indicate MeanDecreaseGini values; the red vertical line marks the feature-selection cutoff of 0.2. (**E**) Intersection of features prioritized by LASSO, random forest, and PPI network connectivity.

To further prioritize features within the 44-gene candidate set, LASSO regression, random forest analysis, and PPI network topology were integrated. Using the lambda.1se criterion, LASSO regression retained *FGFR4* and *PTPRC* (Figure 5A,B). In the random forest analysis, the out-of-bag (OOB) error stabilized as the number of trees increased (Figure 5C). Eleven genes met the MeanDecreaseGini > 0.2 threshold, including *PTPRC* (Figure 5D). Intersection of the LASSO-selected genes, random forest-selected features, and the top 10 PPI nodes by degree identified *PTPRC* as the only shared candidate in the primary 44-gene analysis (Figure 5E). Across GeneCards relevance-score cutoffs from >5 to >10, *PTPRC* was retained by both random forest and PPI analyses. LASSO also retained *PTPRC* at >5, >6, and >7, but not at >8, >9, or >10 (Table S2). *PTPRC* encodes the well-established pan-leukocyte marker CD45 and was therefore interpreted as a leukocyte-associated feature of the bulk-tissue transcriptomic differences between PJS-GAS and PJS-LEGH, rather than indicating a novel tumor-intrinsic mechanism or serving as a stand-alone diagnostic marker.

### 3.6. PJS-Associated GAS Exhibits Higher Immune-Cell-Associated and Immune-Function-Related Transcriptional Scores

**Figure 6:**
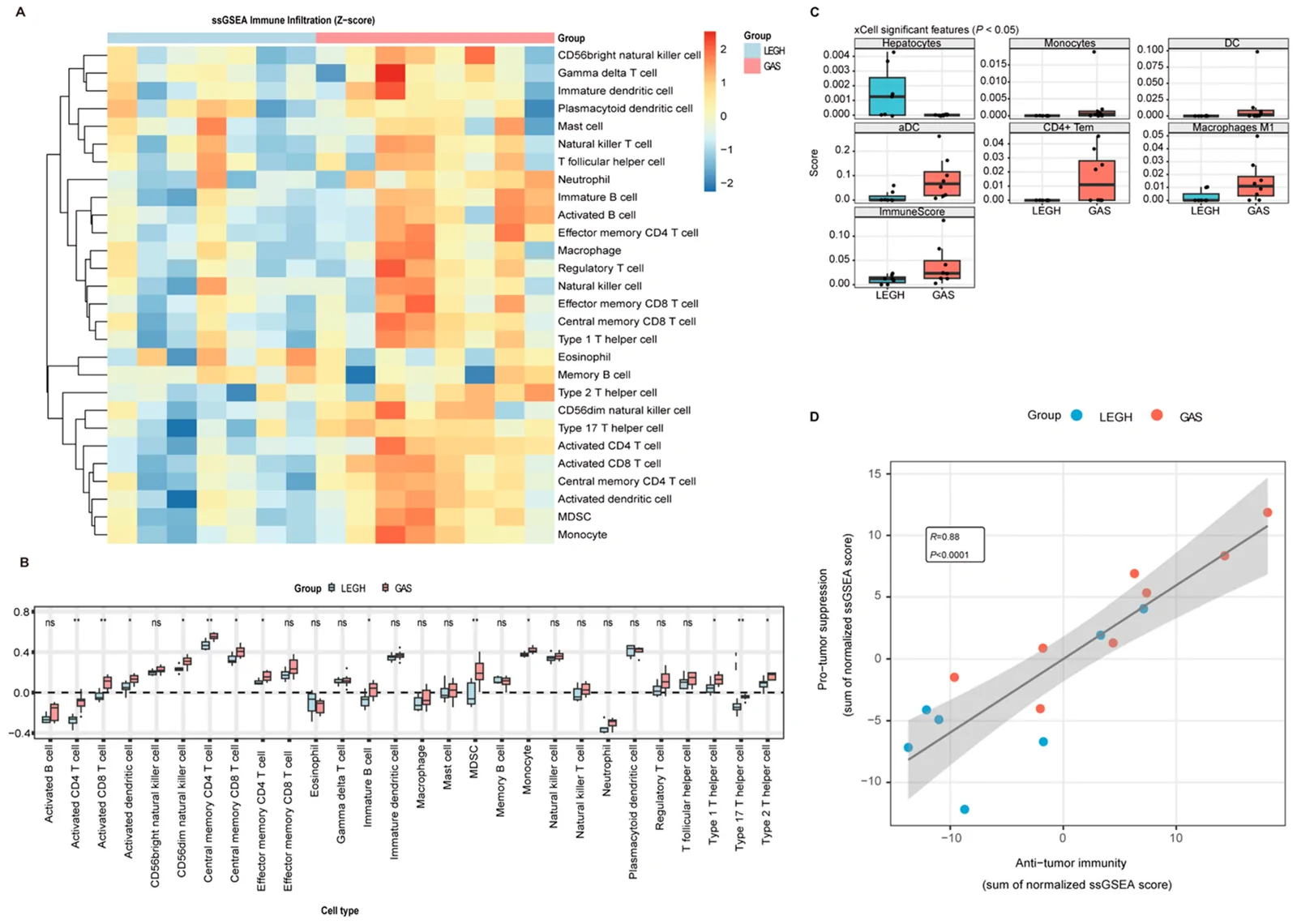
Altered immune infiltration features in PJS-GAS. (**A**) Heatmap of immune-cell-associated enrichment scores estimated by single-sample gene set enrichment analysis (ssGSEA). (**B**) Boxplots comparing ssGSEA-derived immune-cell-associated scores between PJS-LEGH and PJS-GAS. (**C**) xCell-derived immune and microenvironment-related features showing between-group differences at nominal *p* < 0.05. In panels (**B**,**C**), boxes represent the interquartile range, horizontal lines indicate medians, and whiskers extend to the most extreme observations within 1.5 times the interquartile range. In panel (**B**), dots indicate outliers; in panel (**C**), dots represent individual samples. (**D**) Correlation between anti-tumor immune scores and pro-tumor immunosuppressive scores, with point color indicating sample group. The solid line represents the fitted linear regression, and the gray shaded area indicates its 95% confidence interval. Between-group comparisons in panels (**B**,**C**) used two-sided Wilcoxon rank-sum tests. Asterisks in panel (**B**) and feature selection in panel (**C**) are based on unadjusted *p*-values. Adjusted *p*-values for the ssGSEA comparisons are reported in Table S3. ns, not significant; *, *p* < 0.05; **, *p* < 0.01.

Based on the enrichment of immune-inflammatory pathways, we further assessed immune-cell-associated and immune-function-related transcriptional signatures using ssGSEA and xCell. ssGSEA identified increased signatures of activated CD4^+^ and CD8^+^ T cells, dendritic cells, monocytes, myeloid-derived suppressor cells (MDSCs), and T helper (Th) cell subsets (Figure 6A,B). These ssGSEA-derived differences were accompanied by large effect sizes: activated CD4^+^ T cells, Th17 cells, activated CD8^+^ T cells, central memory CD4^+^ T cells, and MDSCs all showed Cliff’s delta ≥ 0.75 with false discovery rate (FDR) < 0.05 (Table S3), indicating substantial between-group differences. Using a complementary approach, xCell also indicated higher enrichment scores for monocytes, dendritic cell populations, CD4^+^ effector memory T cells, and M1 macrophages, together with a higher overall ImmuneScore in PJS-GAS (Figure 6C and Figure S3). Immune functional analysis further showed higher chemokine receptor-related, cytolytic, inflammatory, T-cell co-stimulatory, and T-cell co-inhibitory scores in GAS (Figure S4A,B). Antitumor immune scores were strongly positively correlated with protumor immunosuppressive scores (*R* = 0.88, *p* < 0.0001), with PJS-GAS samples predominantly distributed in the high-score region (Figure 6D). Within the PJS-GAS cohort, no associations between FIGO stage and ssGSEA or xCell enrichment scores remained statistically significant after Benjamini–Hochberg correction (Table S4). Similarly, the supplementary comparison between FIGO stages I–IIA and IIB–IVB identified no significant differences after multiple-testing correction (Table S5). Together, these complementary analyses suggest higher immune-cell-associated signatures in PJS-GAS, accompanied by increased activation- and inhibition-associated transcriptional scores.

### 3.7. PTPRC Expression Is Associated with Inferred Immune-Cell and Immune-Function-Related Scores

**Figure 7:**
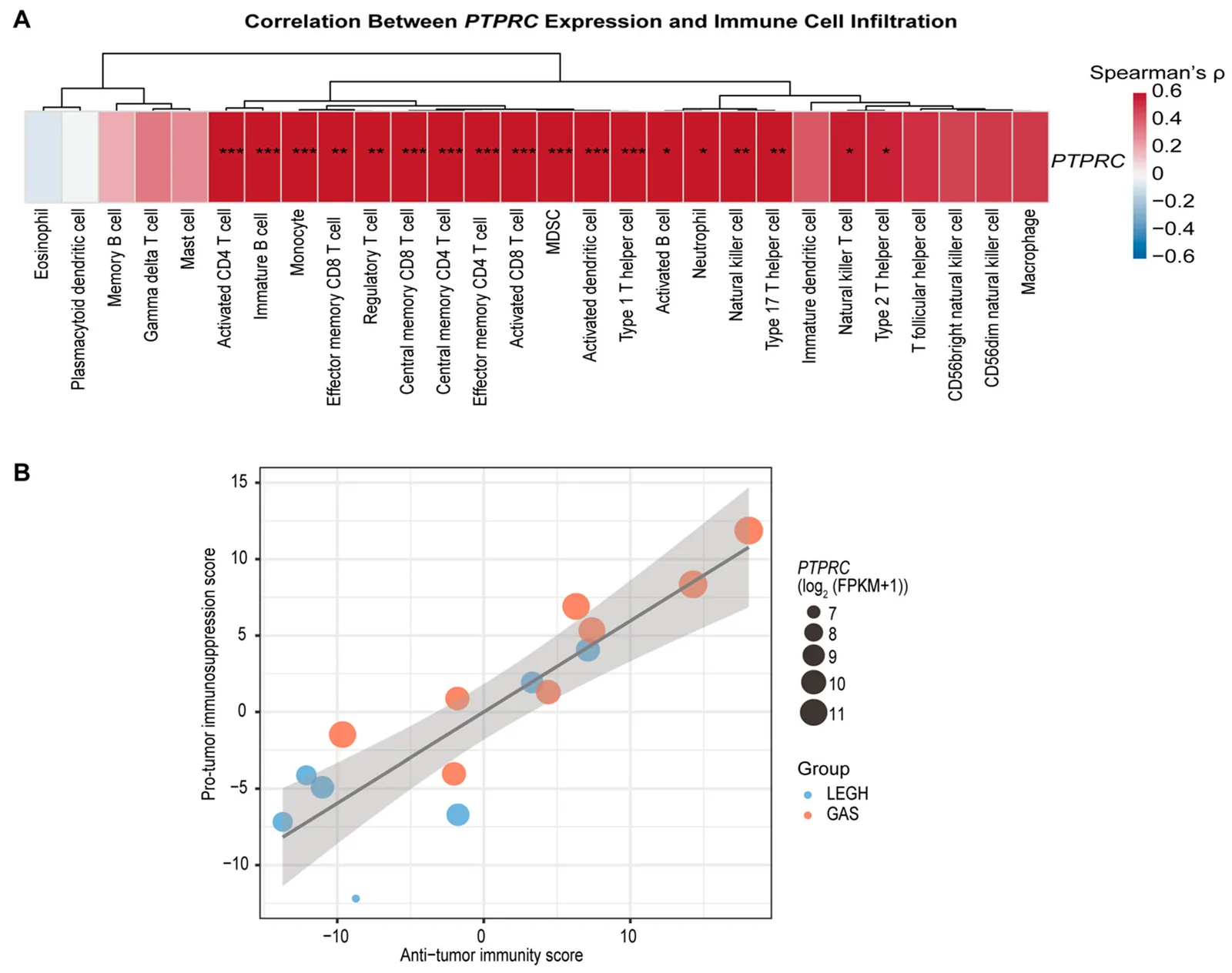
*PTPRC* expression is associated with immune-cell scores and the immune activation–immunoregulation landscape. (**A**) Spearman correlations between *PTPRC* expression and single-sample gene set enrichment analysis (ssGSEA)-derived immune-cell-associated scores. Colors represent Spearman correlation coefficients (ρ), with the color scale saturated at −0.6 and 0.6. Asterisks indicate unadjusted two-sided *p*-values. (**B**) Relationship between antitumor immune and protumor immunoregulatory scores. Each point represents an individual sample; point size represents *PTPRC* expression, and point color indicates group. The solid line represents the fitted linear regression across all samples, and the gray shaded area indicates its 95% confidence interval. *, *p* < 0.05; **, *p* < 0.01; ***, *p* < 0.001.

Given that *PTPRC* encodes CD45 and plays an important role in immune cell signaling and regulation, we further explored its association with immune microenvironmental features. Correlation analysis showed that *PTPRC* expression was positively correlated with multiple ssGSEA-derived immune cell infiltration scores, particularly those for T cell-related subsets, dendritic cells, natural killer (NK) cells, monocytes/macrophages, and MDSCs (Figure 7A). These associations are consistent with *PTPRC* expression reflecting leukocyte-associated variation in the bulk transcriptomic profiles. Samples with higher *PTPRC* expression also tended to have higher scores for both immune activation- and immunosuppression-associated signatures (Figure 7B).

### 3.8. Multiplex Immunofluorescence Supports Transcriptomic Evidence of Increased Immune-Cell Infiltration

**Figure 8:**
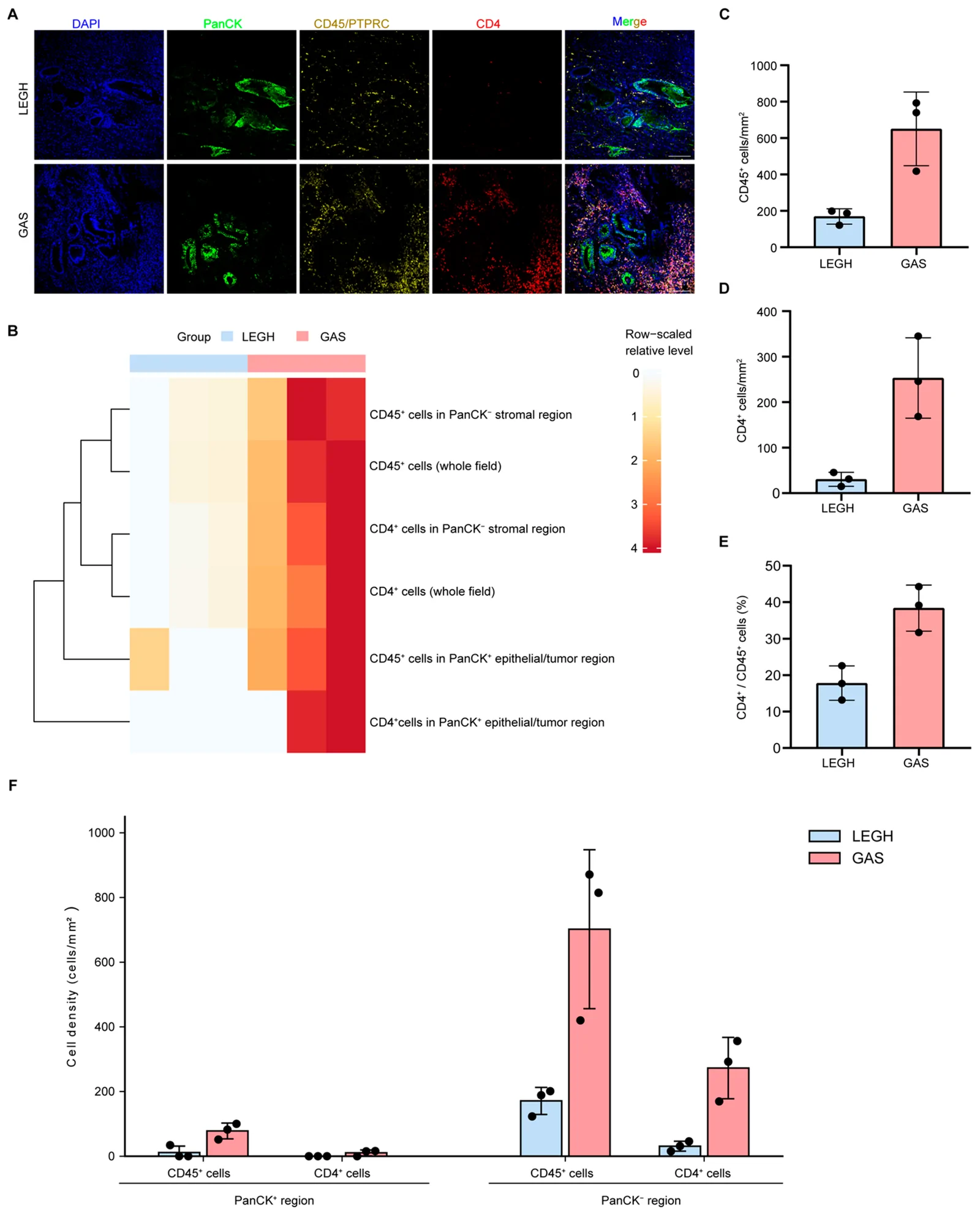
Multiplex immunofluorescence provides preliminary tissue-level support for increased immune-cell infiltration in PJS-GAS. (**A**) Representative PJS-LEGH and PJS-GAS images. Scale bar: 50 μm. (**B**) Patient-level immune-feature heatmap. (**C**) Whole-field CD45^+^ cell density. (**D**) Whole-field CD4^+^ cell density. (**E**) Percentage of CD4^+^ cells among CD45^+^ cells. (**F**) CD45^+^ and CD4^+^ cell densities in pan-cytokeratin-positive (PanCK^+^) epithelial/tumor and PanCK^−^ stromal regions. CD4^+^ cells were defined as CD45^+^CD4^+^ double-positive cells. Three fields of view were averaged to obtain one value per patient (*n* = 3 per group). In (**C**–**F**), dots represent individual patients, bars indicate means, and error bars indicate standard deviations. Normality assessments and exploratory statistical comparisons, including Student’s *t*-tests and exact permutation tests, are provided in Table S6.

To assess the immune microenvironmental alterations inferred from the transcriptomic analyses at the tissue level, multiplex immunofluorescence staining for DAPI, PanCK, CD45/PTPRC, and CD4 was performed in PJS-LEGH and PJS-GAS tissues. CD45/PTPRC was selected to evaluate leukocyte infiltration because *PTPRC* was jointly prioritized in the primary 44-gene analysis and was broadly associated with inferred immune-cell infiltration signatures. CD4 was included because both ssGSEA and xCell indicated increased CD4-related T-cell signatures in PJS-GAS. Representative images and quantitative summaries showed higher CD45^+^ and CD4^+^ cell infiltration in the sampled PJS-GAS tissues than in the PJS-LEGH tissues (Figure 8A,B). Whole-field CD45^+^ and CD4^+^ cell densities, as well as the proportion of CD4^+^ cells among CD45^+^ cells, were higher in the sampled PJS-GAS tissues (Figure 8C–E), suggesting differences in the local immune-cell composition.

Spatial compartment analysis based on PanCK staining further characterized group-wise differences in CD45^+^ and CD4^+^ cell distribution within PanCK-defined epithelial/tumor and stromal compartments. Spatial analysis showed higher observed CD45^+^ cell densities in both PanCK^+^ and PanCK^−^ regions of PJS-GAS tissues, together with higher CD4^+^ cell density in PanCK^−^ regions. CD4^+^ cells were sparse in PanCK^+^ regions (Figure 8F). In PJS-LEGH, Shapiro–Wilk testing detected a departure from normality for CD45^+^ cell density in PanCK^+^ regions, whereas CD4^+^ cell density in these regions was zero in all three patients, precluding normality assessment. Exact permutation *p*-values were 0.10 for six endpoints and 0.40 for CD4^+^ cell density in PanCK^+^ regions (Table S6). These observations provide preliminary descriptive tissue-level support for the immune-cell-associated differences inferred from the transcriptomic analyses.

## 4. Discussion

In this study, we performed the first comparative transcriptomic analysis of PJS-LEGH and PJS-GAS and identified distinct molecular features involving epithelial differentiation, cilium-related processes, and immune-associated programs. By integrating transcriptomic profiling with immune deconvolution analyses and tissue-level assessment, we observed increased immune-cell infiltration signatures in PJS-GAS compared with PJS-LEGH. These findings provide new insights into the molecular characteristics and immune microenvironment of this rare tumor entity.

The DEGs between PJS-GAS and PJS-LEGH were enriched not only in immune-inflammatory pathways but also in cilium movement, axonemal structure, motile cilia, and epithelial differentiation-related processes. Cilia are involved in extracellular signal sensing, tissue development, cell fate determination, and tissue homeostasis [26,27], and ciliary abnormalities have also been implicated in tumor development and progression [28]. Therefore, the altered expression of cilia- and epithelial differentiation-related genes may reflect differences in glandular differentiation status, epithelial structural maintenance, or tissue remodeling between PJS-GAS and PJS-LEGH. However, bulk RNA-seq cannot establish the cellular origin of these signals or demonstrate altered ciliary function. The proposed links to glandular differentiation and tissue remodeling therefore remain hypotheses requiring further investigation.

Immune-associated differences were among the prominent transcriptomic findings of this study. Functional enrichment analyses of DEGs and integrated analyses incorporating DEGs, PJS-GAS associated WGCNA modules, and PJS-associated genes consistently highlighted immune-related biological processes, including leukocyte adhesion, lymphocyte differentiation, T-cell activation, cytokine–cytokine receptor interactions, chemokine signaling, Th17-cell differentiation, and NF-κB signaling. These findings suggest that PJS-GAS and PJS-LEGH differ in immune-related transcriptional programs, including signatures associated with immune-cell infiltration, immune activation, and inhibitory immune regulation. Consistently, ssGSEA and xCell indicated higher immune-cell-related scores in PJS-GAS, including those associated with activated T-cell subsets, dendritic cells, monocyte/macrophage-related populations, and MDSCs. In the sampled tissues, multiplex immunofluorescence showed higher CD45^+^ leukocyte and CD4^+^ cell densities in PJS-GAS, providing preliminary tissue-level support for the immune-cell infiltration patterns inferred from the transcriptomic analyses.

Importantly, the higher overall immune infiltration observed in PJS-GAS compared with PJS-LEGH does not necessarily indicate an effective antitumor immune response. This apparent paradox may be explained by the functional state and cellular composition of the infiltrating immune cells. Previous studies have shown that immune cell composition and immune-checkpoint expression dynamically change during cervical cancer progression [29], and single-cell analyses have revealed that T/NK cells in cervical cancer may shift from cytotoxic states toward exhausted states, whereas cervical adenocarcinoma can exhibit an immunosuppressive microenvironment enriched with regulatory T cells (Tregs) and tumor-promoting neutrophils [30,31]. More directly, a recent multicenter study of GAS demonstrated that GAS has a distinct immunosuppressive tumor microenvironment characterized by relatively sparse T-cell infiltration, macrophage enrichment, particularly M2-like macrophage enrichment, and immunosuppressive tertiary lymphoid structures associated with poor prognosis [16]. The apparently reduced T-cell infiltration in that study likely reflects a different comparator: it characterized GAS relative to HPV-associated cervical carcinomas, against which GAS appears T-cell–poor, whereas we compared PJS-GAS with PJS-LEGH, a benign gastric-type precursor with minimal baseline immune infiltration. The direction of change thus depends on the reference: PJS-GAS is more immune-infiltrated than its benign precursor, yet increased infiltration does not equate to effective antitumor immunity. Consistent with the presence of immunosuppressive features reported in previous studies, our analysis showed that antitumor immune scores were strongly positively correlated with protumor immunosuppressive scores, and PJS-GAS samples were mainly distributed in the high immune activation/high immunosuppression region. These results indicate co-occurring immune activation- and inhibition-associated transcriptional signatures in PJS-GAS, although the functional states of the corresponding immune cells were not directly assessed.

The enrichment of inflammatory and chemokine-related pathways, together with increased *CXCL5* expression, raises the possibility of altered immune-cell recruitment in PJS-GAS, given the roles of chemokines in immune-cell trafficking described in previous studies [32,33]. Chemokine networks regulate the recruitment, localization, and functional states of both antitumor effector cells and immunoregulatory cell populations in tumors. In particular, CXCL5/CXCR2 signaling has been implicated in tumor-associated inflammation, neutrophil recruitment, and myeloid-cell trafficking and may contribute to the accumulation of immunosuppressive myeloid populations, including MDSC-like cells [34,35]. Sustained inflammatory stimulation may also promote T-cell dysfunction or exhaustion, characterized by reduced effector function and increased inhibitory receptor expression [36]. This possibility is consistent with the increased T-cell co-inhibitory signatures observed in PJS-GAS. MDSCs and other immunosuppressive myeloid cells may further promote immune escape by suppressing T-cell and NK-cell activity [37]. Multiplex immunofluorescence showed higher observed CD4^+^ cell density in PanCK^−^ stromal regions of PJS-GAS, while CD4^+^ cells were sparse in PanCK^+^ regions. These observations describe spatial differences in the sampled tissues without establishing a compartment-specific effect. These proposed links between chemokine expression, myeloid-cell recruitment, and T-cell dysfunction remain hypotheses in PJS-GAS. The multiplex immunofluorescence findings provide preliminary tissue-level support for differences in CD45^+^ and CD4^+^ cell density and distribution.

*PTPRC* was jointly prioritized in the primary 44-gene analysis. Because it encodes CD45, a well-established pan-leukocyte marker expressed across hematopoietic populations [38,39], its higher expression in PJS-GAS is best interpreted as a leukocyte-associated component of the bulk-tissue transcriptomic differences rather than a tumor-epithelial driver or stand-alone diagnostic marker. The identification of this established marker, together with its associations with inferred immune-cell scores, provides a biological consistency check for the immune-infiltration findings. Beyond *PTPRC*, other differentially expressed genes may represent additional components of the PJS-GAS molecular phenotype. *CXCL5* was upregulated in PJS-GAS and has been linked to cervical cancer cell proliferation, migration, and tumor growth [40]. *OTX1* has been reported to promote proliferative, migratory, and invasive phenotypes through Wnt-related signaling [41]. The differential expression of these genes reflects broader inflammatory and proliferation-related transcriptional alterations associated with PJS-GAS.

This study has several limitations. First, owing to the rarity of PJS-LEGH and PJS-GAS, only 7 PJS-LEGH and 8 PJS-GAS cases could be included. The small sample size makes the machine-learning analyses susceptible to overfitting, and feature-selection stability under resampling was not assessed; these analyses therefore represent exploratory feature prioritization. Nominal *p*-value screening increases the risk of false-positive inclusion, which is not formally controlled by subsequent intersection or network analyses. GeneCards-based filtering may also exclude biologically relevant genes with limited prior annotation. Differences in age distribution and heterogeneous FIGO stages may confound immune comparisons. Age-associated immune variation has been observed across human tissues [42], and the limited power of the stage-related analyses prevents exclusion of stage-related confounding despite nonsignificant adjusted results. Second, immune-cell composition and functional states were inferred primarily from bulk RNA sequencing, which cannot resolve cell-type-specific expression patterns or tumor–immune interactions. Third, multiplex immunofluorescence was performed in only three cases per group, with two cases per group overlapping with the RNA-sequencing cohort. The small sample size limits both the assessment of normality and statistical power for spatial comparisons. These findings therefore provide preliminary descriptive tissue-level support rather than independent validation. Furthermore, the limited panel comprising PanCK, CD45, and CD4 did not comprehensively characterize immune checkpoint expression or immunosuppressive cell populations. Future studies incorporating larger independent multicenter cohorts, expanded immune marker panels, functional assays, single-cell sequencing, and spatial transcriptomics are needed to further characterize the immune microenvironment and tumor–immune interactions in PJS-GAS.

## 5. Conclusions

PJS-GAS exhibits distinct transcriptomic features compared with PJS-LEGH, involving epithelial differentiation, cilia-related programs, chemokine signaling, and immune microenvironment-related alterations. PJS-GAS shows increased immune-cell-associated transcriptional signatures and activation- and inhibition-associated gene-expression patterns, accompanied by preliminary tissue-level observations of higher CD45^+^ and CD4^+^ cell densities in the sampled tissues. These findings offer new insights into the molecular characteristics and immune microenvironment of this rare malignancy and provide a basis for further investigation of PJS-GAS biology.

## Figures and Tables

**Table 1:** Clinicopathologic characteristics of patients with PJS-LEGH and PJS-GAS.

Patient ID	Age, Years	Clinical Presentation	HPV Status	Germline *STK11* Mutation	FIGO Stage
PJS-associated LEGH (*n* = 7)					
t-PJS-RHH-006	29	Asymptomatic	Negative	Detected	NA
t-PJS-RHH-007	19	Watery vaginal discharge	Negative	Detected	NA
t-PJS-RHH-016	29	Watery vaginal discharge	Negative	Not detected *	NA
t-PJS-RHH-025	38	Asymptomatic	Negative	Detected	NA
t-PJS-RHH-034	18	Watery vaginal discharge	Negative	Detected	NA
t-PJS-RHH-346	30	Watery vaginal discharge	Negative	Detected	NA
t-PJS-RHH-411	24	Watery vaginal discharge	Negative	Detected	NA
PJS-associated GAS (*n* = 8)					
t-PJS-RHH-010	29	Watery vaginal discharge	Negative	Detected	IA1
t-PJS-RHH-044	28	Watery vaginal discharge	Negative	Detected	IB3
t-PJS-RHH-048	44	Watery vaginal discharge	Negative	Detected	IB2
t-PJS-RHH-059	35	Watery vaginal discharge	Negative	Detected	IVB
t-PJS-RHH-129	40	Watery vaginal discharge	Negative	Detected	IIIC1
t-PJS-RHH-480	36	Watery vaginal discharge	Negative	Detected	IIIC1
t-PJS-RHH-486	26	Watery vaginal discharge	Negative	Detected	IB3
t-PJS-RHH-488	26	Watery vaginal discharge	Negative	Detected	IIB

Abbreviations: LEGH, lobular endocervical glandular hyperplasia; GAS, gastric-type endocervical adenocarcinoma; HPV, human papillomavirus; FIGO, International Federation of Gynecology and Obstetrics; NA, not applicable. * Patient t-PJS-RHH-016, in whom no germline *STK11* mutation was detected, was diagnosed with Peutz–Jeghers syndrome based on established clinical diagnostic criteria, namely, the coexistence of characteristic mucocutaneous pigmentation and Peutz–Jeghers-type polyps.

## Data Availability

The data presented in this study are available on request from the corresponding author due to privacy.

## Supplementary Materials

The following supporting information can be downloaded at: https://www.mdpi.com/article/10.3390/biomedicines14102197/s1. Figure S1, Dysregulation of cilia-related genes in PJS-GAS; Figure S2, Dysregulation of epithelial differentiation-related genes in PJS-GAS; Figure S3, Heatmap of xCell-derived immune and microenvironmental features between PJS-LEGH and PJS-GAS samples; Figure S4, PJS-GAS shows higher immune activation- and inhibition-associated transcriptional scores; Table S1, Samples included in multiplex immunofluorescence analysis and their overlap with the RNA-sequencing cohort; Table S2, *PTPRC* prioritization at GeneCards relevance-score cutoffs from >5 to >10; Table S3, Effect sizes for ssGSEA-derived immune-score comparisons between PJS-associated GAS (*n* = 8) and LEGH (*n* = 7); Table S4, Associations between FIGO stage and ssGSEA- and xCell-derived enrichment scores within the PJS-GAS cohort; Table S5, Exploratory comparison of ssGSEA- and xCell-derived enrichment scores between PJS-GAS cases with FIGO stages I–IIA and IIB–IVB; Table S6, Normality assessment and sensitivity analyses of multiplex immunofluorescence measurements.
