# Supplementary material for: Integrated Transcriptomic Analysis Reveals Distinct Molecular and Immune Microenvironment Features of Peutz–Jeghers Syndrome-Associated Lobular Endocervical Glandular Hyperplasia and Gastric-Type Endocervical Adenocarcinoma
Source: Biomedicines. 2026 Sep 28;14(10):2197. doi: 10.3390/biomedicines14102197 (PMC13701942; doi:10.3390/biomedicines14102197)
Supplement: Supplementary file 1 [file biomedicines-14-02197-s001.zip › biomedicines-4526993-supplementary.pdf]

Supplementary Material

Supplementary Figures

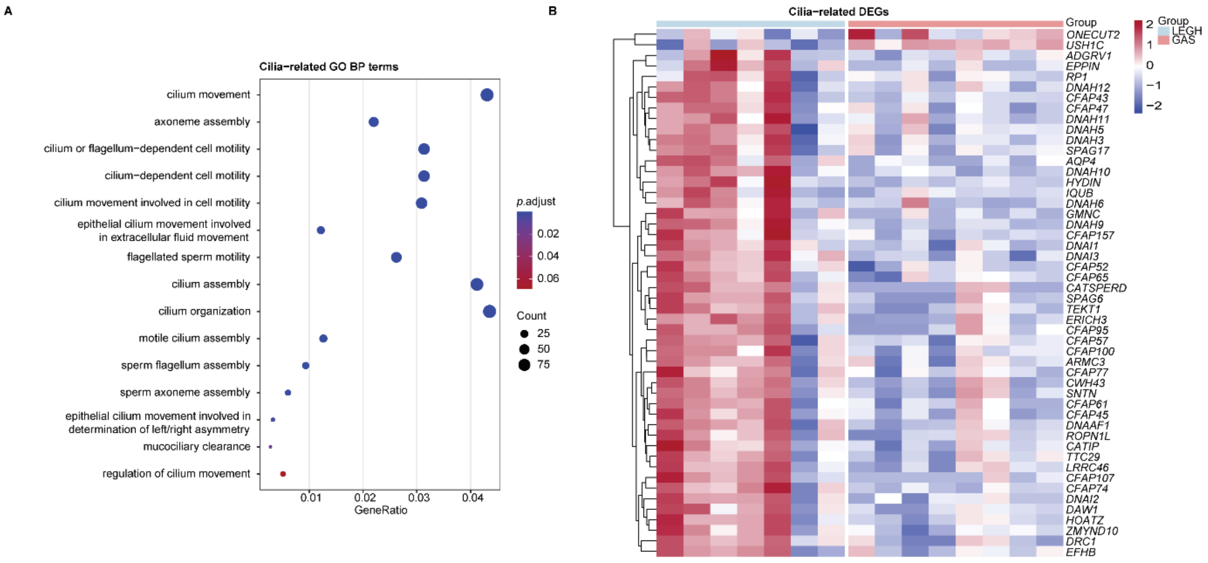

Figure S1. Dysregulation of cilia-related genes in PJS-GAS. (A) Bubble plot of cilia-related GO biological process enrichment terms. (B) Heatmap of representative cilia-related differentially expressed genes between LEGH and GAS.

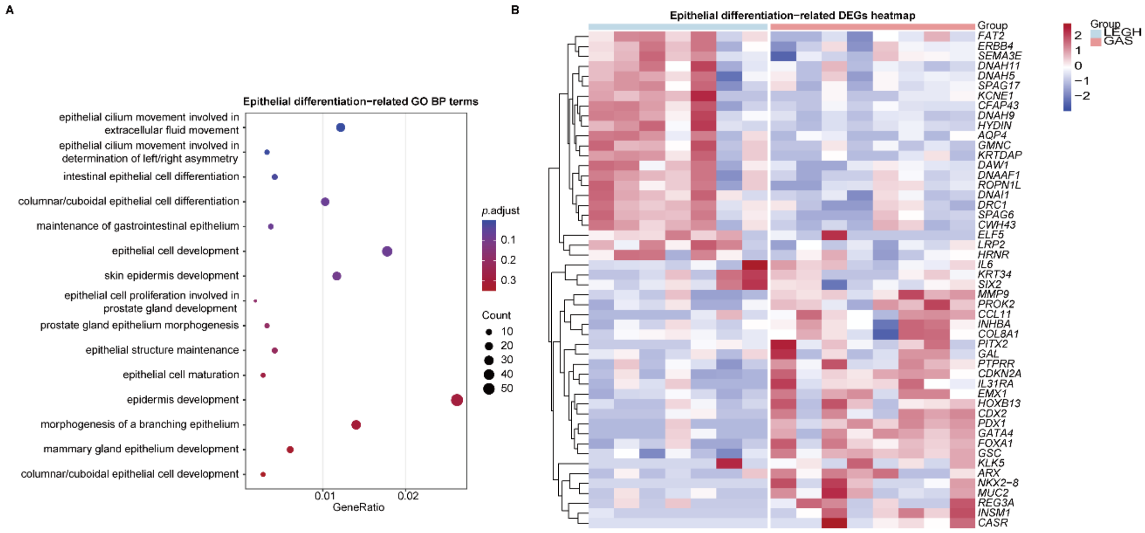

Figure S2. Dysregulation of epithelial differentiation-related genes in PJS-GAS. (A) Bubble plot of epithelial differentiation-related GO biological process enrichment terms. (B) Heatmap of representative epithelial differentiation-related differentially expressed genes between LEGH and GAS.

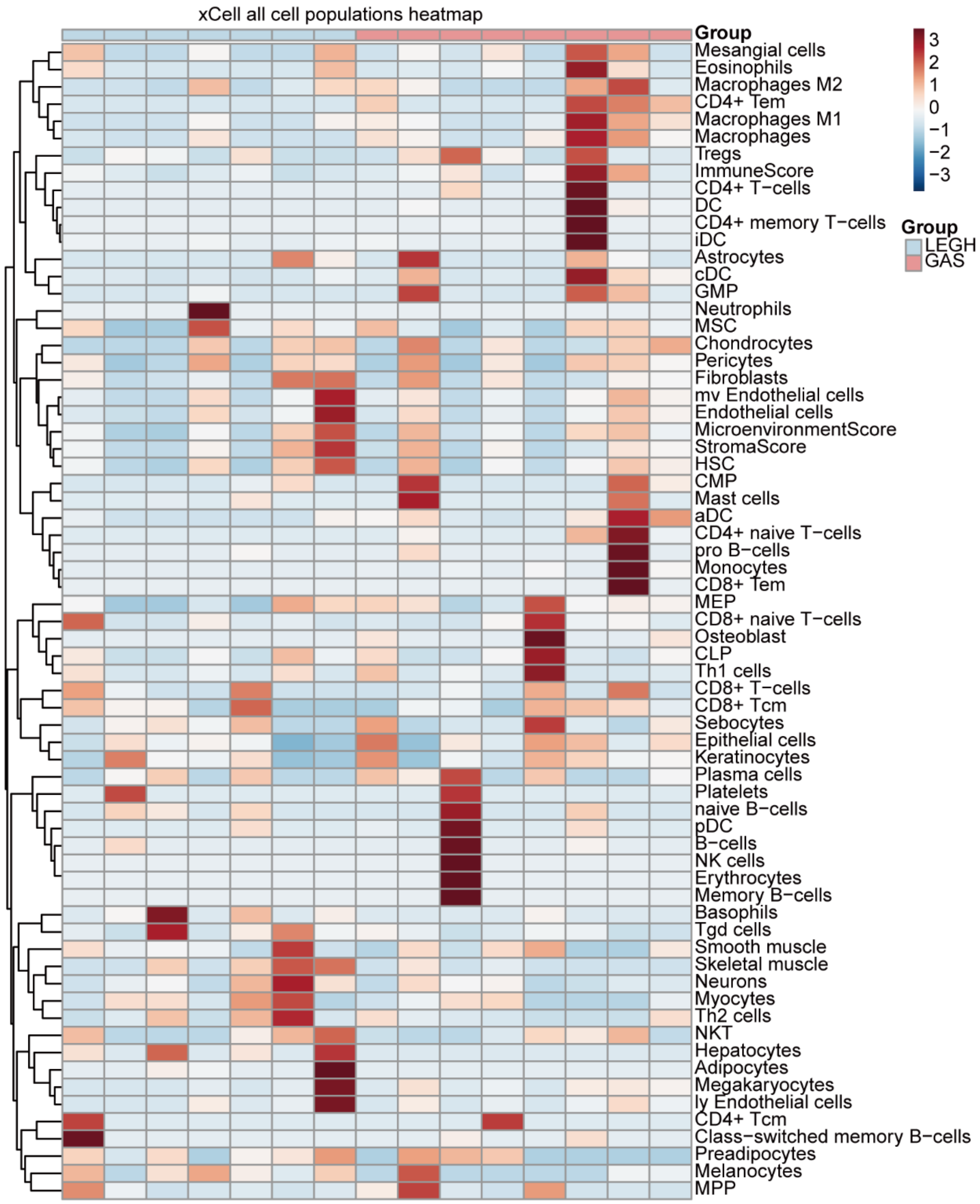

Figure S3. Heatmap of xCell-derived immune and microenvironmental features between PJS-LEGH and PJS-GAS samples.

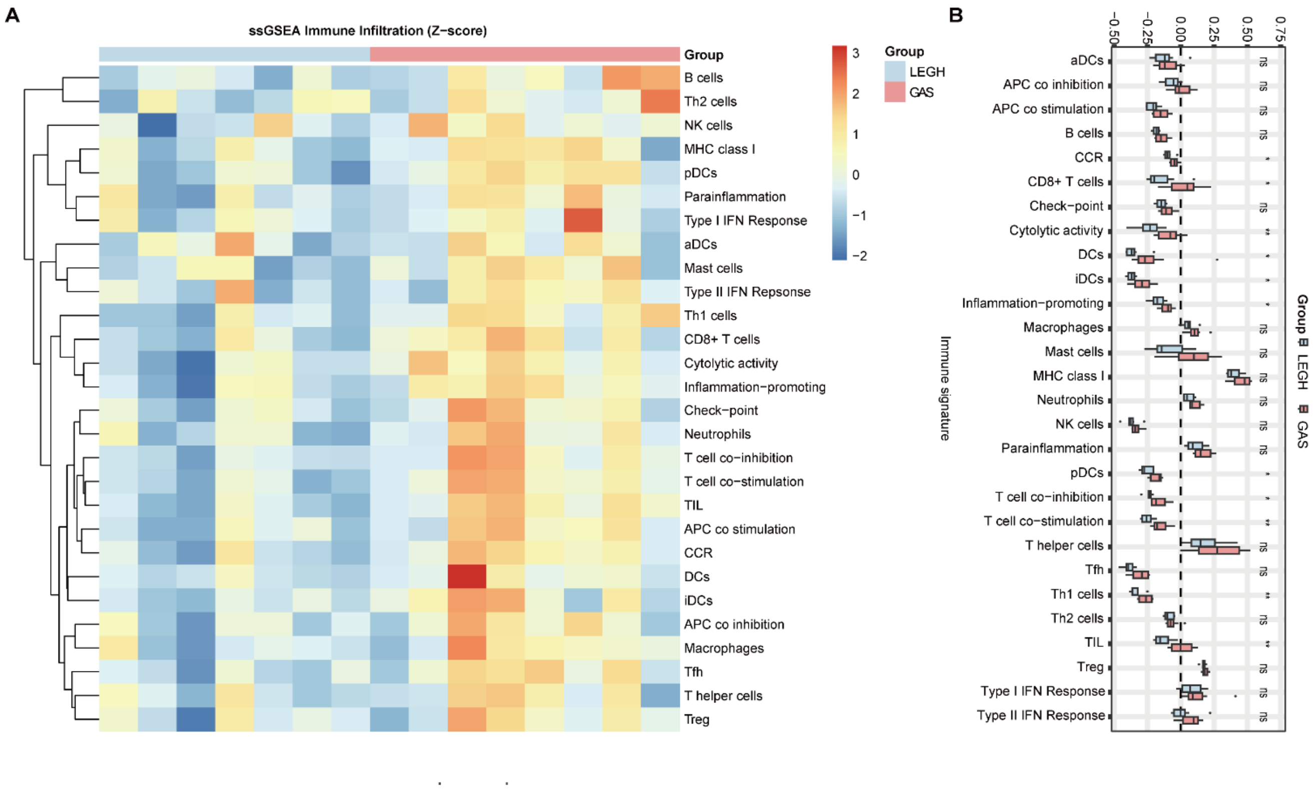

Figure S4. PJS-GAS shows higher immune activation- and inhibition-associated transcriptional scores. (A) Heatmap of ssGSEA-derived immune-function and immune-cell-associated enrichment scores. (B) Boxplots comparing ssGSEA scores between PJS-LEGH and PJS-GAS samples. Dots indicate outliers. Asterisks are based on unadjusted two-sided Wilcoxon rank-sum *p*-values. ns, not significant; *, $p < 0.05$; **, $p < 0.01$.

Table S1. Samples included in multiplex immunofluorescence analysis and their overlap with the RNA-sequencing cohort.

Sample ID	Group	Included in the RNA-seq cohort
t-PJS-RHH-008	PJS-LEGH	No
t-PJS-RHH-346	PJS-LEGH	Yes
t-PJS-RHH-411	PJS-LEGH	Yes
t-PJS-RHH-129	PJS-GAS	Yes
t-PJS-RHH-298	PJS-GAS	No
t-PJS-RHH-480	PJS-GAS	Yes

Table S2. *PTPRC* prioritization at GeneCards relevance-score cutoffs from >5 to >10.

Score cutoff	Candidate genes, n	LASSO	Random forest	PPI	Shared by all three
>5	44	Yes	Yes	Yes	Yes
>6	36	Yes	Yes	Yes	Yes
>7	33	Yes	Yes	Yes	Yes
>8	30	No	Yes	Yes	No
>9	28	No	Yes	Yes	No
>10	25	No	Yes	Yes	No

Yes/No indicates whether *PTPRC* was retained by the corresponding approach. LASSO selection used lambda.1se; random forest selection used MeanDecreaseGini>0.2; PPI selection used the top 10 nodes ranked by degree, with ties resolved alphabetically by gene symbol. The primary analysis used a cutoff of >5.

Table S3. Effect sizes for ssGSEA-derived immune-score comparisons between PJS-associated GAS (n = 8) and LEGH (n = 7).

Immune score	GAS median	LEGH median	Wilcoxon *P*	FDR	Cliff's δ	95% CI	Hedges' g
Activated CD4 T cell	-0.08	-0.263	0.00124	0.0174	0.929	0.679 to 1	2.278
Type 17 T helper cell	-0.04	-0.145	0.00124	0.0174	0.929	0.679 to 1	2.101
Activated CD8 T cell	0.114	-0.052	0.00373	0.0261	0.857	0.536 to 1	2.158
Central memory CD4 T cell	0.553	0.465	0.00373	0.0261	0.857	0.5 to 1	2.012
MDSC	0.192	-0.062	0.00932	0.049	0.786	0.357 to 1	1.574
Activated dendritic cell	0.133	0.05	0.014	0.049	0.75	0.286 to 1	1.392
Central memory CD8 T cell	0.406	0.313	0.014	0.049	0.75	0.286 to 1	1.278
Monocyte	0.412	0.375	0.014	0.049	0.75	0.321 to 1	1.481
Effector memory CD4 T cell	0.154	0.093	0.0205	0.0574	0.714	0.25 to 1	1.32
Type 1 T helper cell	0.13	0.045	0.0205	0.0574	0.714	0.25 to 1	1.279
CD56dim natural killer cell	0.309	0.232	0.0289	0.0736	0.679	0.143 to 1	1.435
Immature B cell	0.044	-0.067	0.0401	0.0864	0.643	0.143 to 1	1.263
Type 2 T helper cell	0.178	0.095	0.0401	0.0864	0.643	0.178 to 1	1.182
Effector memory CD8 T cell	0.233	0.172	0.0939	0.164	0.536	0.036 to 0.964	0.972
Neutrophil	-0.285	-0.352	0.0939	0.164	0.536	-0.107 to 1	0.948

Regulatory T cell	0.107	0.013	0.0939	0.164	0.536	-0.036 to 0.964	0.938
CD56bright natural killer cell	0.22	0.2	0.121	0.199	0.5	-0.071 to 0.929	0.842
Activated B cell	-0.151	-0.269	0.152	0.236	0.464	-0.108 to 0.964	1.025
Natural killer T cell	0.026	-0.044	0.232	0.325	0.393	-0.179 to 0.858	0.624
Natural killer cell	0.359	0.339	0.232	0.325	0.393	-0.179 to 0.857	0.48
Immature dendritic cell	0.367	0.35	0.336	0.428	0.321	-0.286 to 0.857	0.505
T follicular helper cell	0.149	0.102	0.336	0.428	0.321	-0.286 to 0.893	0.503
Eosinophil	-0.106	-0.065	0.397	0.483	-0.286	-0.893 to 0.357	-0.321
Macrophage	-0.077	-0.127	0.463	0.54	0.25	-0.357 to 0.786	0.531
Gamma delta T cell	0.116	0.101	0.613	0.687	0.179	-0.429 to 0.786	0.202
Plasmacytoid dendritic cell	0.43	0.435	0.694	0.747	-0.143	-0.75 to 0.536	-0.002
Mast cell	0.024	-0.027	0.867	0.899	0.071	-0.5 to 0.714	0.101
Memory B cell	0.117	0.102	1	1	0	-0.643 to 0.643	-0.386

Groups were compared using the Wilcoxon rank-sum test; *P* values were adjusted using the Benjamini–Hochberg method (FDR). Effect sizes are reported as Cliff's δ (with bootstrap 95% CI) and Hedges' g (bias-corrected standardized mean difference). $|\delta| \geq 0.474$ and $|g| \geq 0.8$ are conventionally regarded as large effects. Rows are sorted by ascending Wilcoxon *P* value.

Table S4. Associations between FIGO stage and ssGSEA- and xCell-derived enrichment scores within the PJS-GAS cohort.

Method	Feature	Spearman's ρ	Exact *P* value	BH-adjusted *P* value
ssGSEA	Memory B cell	-0.807	0.0216	0.6056
ssGSEA	Activated B cell	-0.590	0.1333	0.6131
ssGSEA	Immature B cell	-0.554	0.1627	0.6131
ssGSEA	Type 2 T helper cell	-0.554	0.1627	0.6131
ssGSEA	Monocyte	0.542	0.1698	0.6131
ssGSEA	Eosinophil	-0.518	0.1954	0.6131
ssGSEA	Activated CD8 T cell	0.482	0.2327	0.6131
ssGSEA	Activated dendritic cell	0.482	0.2327	0.6131
ssGSEA	CD56dim natural killer cell	0.482	0.2327	0.6131
ssGSEA	Type 17 T helper cell	0.482	0.2327	0.6131
ssGSEA	Immature dendritic cell	0.470	0.2409	0.6131
ssGSEA	CD56bright natural killer cell	0.434	0.2847	0.6644
ssGSEA	Effector memory CD4 T cell	-0.410	0.3183	0.6855
ssGSEA	Plasmacytoid dendritic cell	0.374	0.3633	0.6993
ssGSEA	Gamma delta T cell	0.361	0.3746	0.6993
ssGSEA	Central memory CD4 T cell	0.313	0.4484	0.7847
ssGSEA	Macrophage	0.253	0.5409	0.8775
ssGSEA	Central memory CD8 T cell	0.241	0.5641	0.8775
ssGSEA	MDSC	0.217	0.6062	0.8933

ssGSEA	T follicular helper cell	-0.181	0.6690	0.9367
ssGSEA	Effector memory CD8 T cell	-0.133	0.7560	0.9970
ssGSEA	Activated CD4 T cell	0.120	0.7833	0.9970
ssGSEA	Natural killer T cell	0.072	0.8708	1.0000
ssGSEA	Type 1 T helper cell	-0.072	0.8708	1.0000
ssGSEA	Neutrophil	-0.048	0.9240	1.0000
ssGSEA	Natural killer cell	0.036	0.9401	1.0000
ssGSEA	Mast cell	0.024	0.9667	1.0000
ssGSEA	Regulatory T cell	0.000	1.0000	1.0000
xCell	Monocytes	0.807	0.0216	0.9174
xCell	Preadipocytes	-0.745	0.0452	0.9174
xCell	Tregs	-0.723	0.0504	0.9174
xCell	CLP	0.699	0.0635	0.9174
xCell	Neurons	-0.627	0.1062	0.9174
xCell	Osteoblast	0.617	0.1122	0.9174
xCell	CD8+ naive T-cells	0.602	0.1202	0.9174
xCell	NKT	0.554	0.1627	0.9174
xCell	CD4+ Tem	0.542	0.1698	0.9174
xCell	CD4+ Tcm	-0.536	0.1774	0.9174
xCell	CD8+ Tem	0.531	0.1818	0.9174
xCell	Sebocytes	0.527	0.1839	0.9174

xCell	Myocytes	-0.506	0.2044	0.9174
xCell	Basophils	0.506	0.2077	0.9174
xCell	Keratinocytes	0.482	0.2327	0.9174
xCell	CD4+ T-cells	-0.444	0.2622	0.9174
xCell	Th1 cells	0.418	0.3029	0.9174
xCell	Epithelial cells	0.410	0.3183	0.9174
xCell	Adipocytes	-0.394	0.3296	0.9174
xCell	Macrophages M1	0.374	0.3633	0.9174
xCell	MEP	0.361	0.3746	0.9174
xCell	iDC	-0.361	0.3746	0.9174
xCell	Erythrocytes	-0.417	0.3750	0.9174
xCell	naive B-cells	-0.346	0.4015	0.9174
xCell	Mast cells	-0.345	0.4048	0.9174
xCell	CD4+ memory T-cells	-0.337	0.4169	0.9174
xCell	Fibroblasts	-0.337	0.4169	0.9174
xCell	Mesangial cells	-0.337	0.4169	0.9174
xCell	Microenvironment Score	-0.337	0.4169	0.9174
xCell	GMP	-0.309	0.4545	0.9174
xCell	Platelets	-0.296	0.4607	0.9174
xCell	MPP	-0.301	0.4688	0.9174
xCell	pDC	-0.291	0.4773	0.9174

xCell	aDC	0.289	0.4851	0.9174
xCell	Astrocytes	-0.272	0.5149	0.9174
xCell	MSC	0.265	0.5282	0.9174
xCell	Megakaryocytes	-0.265	0.5282	0.9174
xCell	Pericytes	-0.265	0.5282	0.9174
xCell	CMP	0.224	0.5864	0.9413
xCell	Macrophages	0.229	0.5867	0.9413
xCell	B-cells	-0.200	0.6316	0.9413
xCell	Stroma Score	-0.193	0.6538	0.9413
xCell	CD4+ naive T-cells	0.185	0.6574	0.9413
xCell	DC	-0.181	0.6690	0.9413
xCell	Eosinophils	-0.176	0.6724	0.9413
xCell	Memory B-cells	-0.173	0.6833	0.9413
xCell	Neutrophils	0.158	0.7082	0.9413
xCell	mv Endothelial cells	0.157	0.7157	0.9413
xCell	NK cells	-0.141	0.7321	0.9413
xCell	Skeletal muscle	-0.139	0.7401	0.9413
xCell	Immune Score	-0.133	0.7560	0.9413
xCell	Class-switched memory B-cells	-0.122	0.7690	0.9413
xCell	Plasma cells	-0.121	0.7739	0.9413
xCell	pro B-cells	-0.103	0.8036	0.9413

xCell	CD8+ T-cells	0.096	0.8272	0.9413
xCell	Chondrocytes	0.096	0.8272	0.9413
xCell	Macrophages M2	0.096	0.8272	0.9413
xCell	cDC	-0.096	0.8272	0.9413
xCell	Th2 cells	-0.072	0.8708	0.9549
xCell	CD8+ Tcm	0.060	0.8946	0.9549
xCell	HSC	0.060	0.8946	0.9549
xCell	Melanocytes	0.062	0.8970	0.9549
xCell	Endothelial cells	0.048	0.9240	0.9680
xCell	Smooth muscle	-0.036	0.9388	0.9681
xCell	Tgd cells	0.006	0.9929	0.9929
xCell	ly Endothelial cells	-0.006	0.9929	0.9929
xCell	Hepatocytes	—	—	—

Analyses included eight PJS-GAS cases. FIGO stage was treated as an ordered categorical variable (IA1 < IB2 < IB3 < IIB < IIIC1 < IVB). Two-sided exact permutation *P* values were calculated by enumerating all 10,080 distinct arrangements of stage ranks and adjusted using the Benjamini–Hochberg method separately for the ssGSEA and xCell score panels (28 and 66 evaluable scores, respectively). The Hepatocytes score was constant across the eight PJS-GAS samples; its correlation could not be calculated, and the corresponding entries are indicated by "—". Features are ordered by ascending unadjusted *P* value within each method.

Table S5. Exploratory comparison of ssGSEA- and xCell-derived enrichment scores between PJS-GAS cases with FIGO stages I–IIA and IIB–IVB.

**Method**	**Feature**	**FIGO I–IIA median**	**FIGO IIB–IVB median**	**Cliff's δ**	**Exact *P* value**	**BH-adjusted *P* value**
ssGSEA	Memory B cell	0.1473	0.0571	-1.000	0.0286	0.8000
ssGSEA	Activated B cell	-0.1152	-0.2216	-0.750	0.1143	0.8727
ssGSEA	Eosinophil	-0.0839	-0.2045	-0.750	0.1143	0.8727
ssGSEA	CD56bright natural killer cell	0.2048	0.2360	0.625	0.2000	0.8727
ssGSEA	Activated dendritic cell	0.0975	0.1533	0.500	0.3429	0.8727
ssGSEA	Gamma delta T cell	0.0857	0.1259	0.500	0.3429	0.8727
ssGSEA	Immature B cell	0.0886	-0.0106	-0.500	0.3429	0.8727
ssGSEA	Immature dendritic cell	0.3414	0.3734	0.500	0.3429	0.8727
ssGSEA	Monocyte	0.3964	0.4253	0.500	0.3429	0.8727
ssGSEA	Plasmacytoid dendritic cell	0.4132	0.4346	0.500	0.3429	0.8727
ssGSEA	Type 2 T helper cell	0.1832	0.1399	-0.500	0.3429	0.8727
ssGSEA	Activated CD8 T cell	0.0703	0.1372	0.375	0.4857	0.9067
ssGSEA	CD56dim natural killer cell	0.2738	0.3301	0.375	0.4857	0.9067
ssGSEA	Effector memory CD4 T cell	0.1815	0.1380	-0.375	0.4857	0.9067
ssGSEA	T follicular helper cell	0.1752	0.1182	-0.375	0.4857	0.9067
ssGSEA	Effector memory CD8 T cell	0.2654	0.2204	-0.250	0.6857	0.9143
ssGSEA	MDSC	0.1645	0.1917	0.250	0.6857	0.9143
ssGSEA	Macrophage	-0.0752	-0.0772	0.250	0.6857	0.9143

ssGSEA	Neutrophil	-0.3107	-0.2826	0.250	0.6857	0.9143
ssGSEA	Type 1 T helper cell	0.1389	0.1214	-0.250	0.6857	0.9143
ssGSEA	Type 17 T helper cell	-0.0485	-0.0401	0.250	0.6857	0.9143
ssGSEA	Activated CD4 T cell	-0.1006	-0.0690	0.125	0.8857	1.0000
ssGSEA	Central memory CD4 T cell	0.5464	0.5637	0.125	0.8857	1.0000
ssGSEA	Central memory CD8 T cell	0.3902	0.4138	0.125	0.8857	1.0000
ssGSEA	Mast cell	0.0264	0.0240	0.000	1.0000	1.0000
ssGSEA	Natural killer T cell	0.0357	0.0257	0.000	1.0000	1.0000
ssGSEA	Natural killer cell	0.3563	0.3589	0.000	1.0000	1.0000
ssGSEA	Regulatory T cell	0.1171	0.1066	0.000	1.0000	1.0000
xCell	Tregs	0.0339	0.0028	-1.000	0.0286	0.7882
xCell	Monocytes	7.31e-18	0.0015	0.875	0.0571	0.7882
xCell	Osteoblast	0.0000	0.0092	0.875	0.0571	0.7882
xCell	CLP	0.0036	0.0208	0.750	0.1143	0.7882
xCell	MEP	0.0116	0.0210	0.750	0.1143	0.7882
xCell	Neurons	0.0029	7.00e-04	-0.750	0.1143	0.7882
xCell	CD4+ Tcm	2.06e-18	0.0000	-0.688	0.1429	0.7882
xCell	Preadipocytes	0.0574	0.0000	-0.688	0.1429	0.7882
xCell	Adipocytes	1.19e-19	6.55e-22	-0.625	0.1714	0.7882
xCell	Th1 cells	5.53e-04	0.0104	0.625	0.1714	0.7882
xCell	CD4+ T-cells	0.0012	7.75e-19	-0.625	0.2000	0.7882

xCell	CD4+ memory T-cells	2.00e-04	1.84e-19	-0.625	0.2000	0.7882
xCell	CD8+ Tem	5.35e-20	6.70e-19	0.625	0.2000	0.7882
xCell	Keratinocytes	0.0105	0.0214	0.625	0.2000	0.7882
xCell	Mesangial cells	0.0115	0.0015	-0.625	0.2000	0.7882
xCell	Myocytes	0.0070	9.03e-04	-0.625	0.2000	0.7882
xCell	naive B-cells	0.0040	4.03e-19	-0.625	0.2000	0.7882
xCell	Sebocytes	0.0028	0.0136	0.562	0.2286	0.8344
xCell	B-cells	0.0028	2.12e-19	-0.500	0.3143	0.8344
xCell	CMP	1.93e-19	0.0012	0.500	0.3143	0.8344
xCell	Eosinophils	0.0024	1.99e-05	-0.500	0.3143	0.8344
xCell	CD4+ Tem	4.32e-19	0.0235	0.500	0.3429	0.8344
xCell	CD8+ naive T-cells	0.0027	0.0052	0.500	0.3429	0.8344
xCell	Epithelial cells	0.1370	0.2326	0.500	0.3429	0.8344
xCell	Fibroblasts	0.0759	0.0443	-0.500	0.3429	0.8344
xCell	Microenvironment Score	0.1280	0.0576	-0.500	0.3429	0.8344
xCell	NKT	0.0025	0.0092	0.500	0.3429	0.8344
xCell	Neutrophils	7.85e-20	1.41e-19	0.375	0.4286	0.8344
xCell	Basophils	1.20e-18	3.31e-18	0.375	0.4857	0.8344
xCell	DC	0.0037	0.0021	-0.375	0.4857	0.8344
xCell	GMP	0.0142	7.27e-04	-0.375	0.4857	0.8344
xCell	MSC	0.0524	0.0986	0.375	0.4857	0.8344

xCell	Macrophages	0.0064	0.0167	0.375	0.4857	0.8344
xCell	Macrophages M1	0.0045	0.0143	0.375	0.4857	0.8344
xCell	Megakaryocytes	0.0011	6.88e-04	-0.375	0.4857	0.8344
xCell	Memory B-cells	1.96e-18	9.90e-19	-0.375	0.4857	0.8344
xCell	Pericytes	0.1633	0.0616	-0.375	0.4857	0.8344
xCell	aDC	0.0493	0.1080	0.375	0.4857	0.8344
xCell	iDC	0.0294	0.0161	-0.375	0.4857	0.8344
xCell	pDC	9.48e-04	1.48e-18	-0.312	0.5429	0.9093
xCell	Plasma cells	0.0093	0.0220	0.250	0.6000	0.9571
xCell	Class-switched memory B-cells	7.69e-04	1.99e-20	-0.250	0.6571	0.9571
xCell	Melanocytes	9.65e-21	4.40e-04	0.250	0.6571	0.9571
xCell	Platelets	1.08e-18	5.65e-19	-0.250	0.6571	0.9571
xCell	CD8+ T-cells	0.0014	0.0105	0.250	0.6857	0.9571
xCell	Macrophages M2	0.0025	0.0053	0.250	0.6857	0.9571
xCell	Stroma Score	0.0603	0.0445	-0.250	0.6857	0.9571
xCell	cDC	0.0589	0.0253	-0.250	0.6857	0.9571
xCell	Mast cells	4.21e-19	0.0000	-0.250	0.7143	0.9767
xCell	Tgd cells	8.69e-19	8.07e-19	0.188	0.7714	1.0000
xCell	Immune Score	0.0320	0.0187	-0.125	0.8857	1.0000
xCell	MPP	5.84e-19	2.48e-18	0.125	0.8857	1.0000
xCell	mv Endothelial cells	0.0407	0.0392	0.125	0.8857	1.0000

xCell	Astrocytes	0.0112	1.52e-18	-0.125	0.9143	1.0000
xCell	CD4+ naive T-cells	0.0032	0.0012	0.125	0.9143	1.0000
xCell	Smooth muscle	0.0791	0.0614	-0.062	0.9714	1.0000
xCell	ly Endothelial cells	0.0161	0.0094	-0.062	0.9714	1.0000
xCell	CD8+ Tcm	0.0114	0.0150	0.000	1.0000	1.0000
xCell	Chondrocytes	0.0208	0.0249	0.000	1.0000	1.0000
xCell	Endothelial cells	0.0448	0.0447	0.000	1.0000	1.0000
xCell	Erythrocytes	0.0000	0.0000	-0.250	1.0000	1.0000
xCell	HSC	0.1572	0.1125	0.000	1.0000	1.0000
xCell	Hepatocytes	0.0000	0.0000	0.000	1.0000	1.0000
xCell	NK cells	3.72e-19	3.89e-19	0.000	1.0000	1.0000
xCell	Skeletal muscle	3.08e-04	2.98e-18	0.000	1.0000	1.0000
xCell	Th2 cells	0.0026	0.0066	0.000	1.0000	1.0000
xCell	pro B-cells	0.0000	0.0000	0.062	1.0000	1.0000

Each group included four PJS-GAS cases. Scores were compared using two-sided exact permutation Mann–Whitney tests, enumerating all 70 group assignments and accounting for tied ranks. Positive Cliff's δ values indicate higher scores in the FIGO IIB–IVB group. *P* values were adjusted using the Benjamini–Hochberg method separately for the ssGSEA and xCell score panels (28 and 67 scores, respectively). Constant scores were retained with $P = 1$ and Cliff's $\delta = 0$. Small nonzero medians are displayed in scientific notation to distinguish them from zero. Features are ordered by ascending unadjusted *P* value within each method.

Table S6. Normality assessment and sensitivity analyses of multiplex immunofluorescence measurements.

Measurement	Shapiro-Wilk PJS-LEGH	Shapiro-Wilk PJS-GAS	Student's t-test	Exact permutation *P*
Whole-field CD45+ density	0.2645	0.2518	0.0158	0.1000
Whole-field CD45+CD4+ density	0.9399	0.8615	0.0127	0.1000
CD45+CD4+ / CD45+ (%)	0.9585	0.7999	0.0106	0.1000
PanCK+ region: CD45+ density	<0.001	0.7139	0.0216	0.1000
PanCK- region: CD45+ density	0.2835	0.2196	0.0210	0.1000
PanCK+ region: CD45+CD4+ density	N/A	0.0936	0.1164	0.4000
PanCK- region: CD45+CD4+ density	0.9591	0.6568	0.0121	0.1000

Values are unadjusted *P* values. Measurements from three fields were averaged per patient (n=3 per group). Densities are expressed as cells/mm², and percentages are averages of field-level percentages. Shapiro–Wilk tests were performed within each group; N/A indicates that all values were zero. Between-group comparisons used two-sided Student's t-tests assuming equal variances and exact permutation tests based on the absolute difference in patient-level means. The minimum attainable two-sided exact *P* value was 0.10. Analyses were exploratory.
